# Brain gray matter astroglia-specific connexin 43 ablation attenuates spinal cord inflammatory demyelination

**DOI:** 10.1186/s12974-021-02176-1

**Published:** 2021-06-05

**Authors:** Hayato Une, Ryo Yamasaki, Satoshi Nagata, Hiroo Yamaguchi, Yuko Nakamuta, Ulfa Camelia Indiasari, Yiwen Cui, Koji Shinoda, Katsuhisa Masaki, Magdalena Götz, Jun-ichi Kira

**Affiliations:** 1 Department of Neurology, Neurological Institute, Graduate School of Medical Sciences, Kyushu University, 3-1-1, Maidashi, Higashi-ku, Fukuoka, 812-8582 Japan; 2 Physiological Genomics, Biomedical Center, Ludwig-Maximilians University Munich, Munich, Germany; 3 Translational Neuroscience Center, Graduate School of Medicine, and School of Pharmacy at Fukuoka, International University of Health and Welfare, 137-1 Enokizu, Ookawa, Fukuoka, 831-8501 Japan; 4 Department of Neurology, Brain and Nerve Center, Fukuoka Central Hospital, International University of Health and Welfare, 2-6-11 Yakuin, Chuo-ku, Fukuoka, 810-0022 Japan

**Keywords:** Experimental autoimmune encephalomyelitis, Connexin 43, Multiple sclerosis, Microglia, Astroglia, Chemokine, Cytokine

## Abstract

**Background:**

Brain astroglia are activated preceding the onset of experimental autoimmune encephalomyelitis (EAE), an animal model of multiple sclerosis (MS). We characterized the effects of brain astroglia on spinal cord inflammation, focusing on astroglial connexin (Cx)43, because we recently reported that Cx43 has a critical role in regulating neuroinflammation.

**Methods:**

Because glutamate aspartate transporter (GLAST)^+^ astroglia are enriched in the brain gray matter, we generated *Cx43*^*fl/fl*^;*GLAST*-*CreER*^*T2/+*^ mice that were brain gray matter astroglia-specific *Cx43* conditional knockouts (*Cx43* icKO). EAE was induced by immunization with myelin oligodendroglia glycoprotein (MOG) _35–55_ peptide 10 days after tamoxifen injection. *Cx43*^*fl/fl*^ mice were used as controls.

**Results:**

Acute and chronic EAE signs were significantly milder in *Cx43* icKO mice than in controls whereas splenocyte MOG-specific responses were unaltered. Histologically, *Cx43* icKO mice showed significantly less demyelination and fewer CD45^+^ infiltrating immunocytes, including F4/80^+^ macrophages, and Iba1^+^ microglia in the spinal cord than controls. Microarray analysis of the whole cerebellum revealed marked upregulation of anti-inflammatory A2-specific astroglia gene sets in the pre-immunized phase and decreased proinflammatory A1-specific and pan-reactive astroglial gene expression in the onset phase in *Cx43* icKO mice compared with controls. Astroglia expressing C3, a representative A1 marker, were significantly decreased in the cerebrum, cerebellum, and spinal cord of *Cx43* icKO mice compared with controls in the peak phase. Isolated *Cx43* icKO spinal microglia showed more anti-inflammatory and less proinflammatory gene expression than control microglia in the pre-immunized phase. In particular, microglial expression of *Ccl2*, *Ccl5*, *Ccl7*, and *Ccl8* in the pre-immunized phase and of *Cxcl9* at the peak phase was lower in *Cx43* icKO than in controls. Spinal microglia circularity was significantly lower in *Cx43* icKO than in controls in the peak phase. Significantly lower interleukin (IL)-6, interferon-γ, and IL-10 levels were present in cerebrospinal fluid from *Cx43* icKO mice in the onset phase compared with controls.

**Conclusions:**

The ablation of *Cx43* in brain gray matter astroglia attenuates EAE by promoting astroglia toward an anti-inflammatory phenotype and suppressing proinflammatory activation of spinal microglia partly through depressed cerebrospinal fluid proinflammatory cytokine/chemokine levels. Brain astroglial Cx43 might be a novel therapeutic target for MS.

**Supplementary Information:**

The online version contains supplementary material available at 10.1186/s12974-021-02176-1.

## Background

Multiple sclerosis (MS) is an inflammatory demyelinating disease of the central nervous system (CNS). Demyelination is accompanied by activation of astroglia in acute and chronic MS lesions [1]. Acute MS lesions contain numerous hypertrophic astrocytes that secrete multiple proinflammatory cytokines and chemokines, thereby augmenting neuroinflammation, as well as various growth factors that promote oligodendrocytes to form myelin by influencing oligodendrocyte progenitor cells [2, 3]. In chronic MS lesions, astrogliotic scars are formed, which may prevent axonal growth and tissue repair. However, the ablation of proliferating astroglia exacerbates experimental autoimmune encephalomyelitis (EAE), an animal model of MS that is associated with a massive infiltration of macrophages and T cells [4], which indicates critical roles of astroglia in preventing the expansion of neuroinflammation. Therefore, astroglia exert proinflammatory and neuroprotective effects on MS pathology.

We and others reported marked alterations of glial connexins (Cxs) in autopsied MS lesions. A previous study reported the loss of astroglial Cxs 30 and 43 and oligodendroglial Cxs 32 and 47 in acute plaques, and unchanged Cxs 32 and 47, and extensively upregulated Cx43 reflecting astrogliosis in chronic plaques [5, 6]. Cxs, transmembrane proteins that form gap junction (GJ) channels that allow the intercellular exchange of ions, secondary messengers, and energy sources [7, 8], have crucial roles in maintaining metabolic homeostasis of the brain [9, 10]. Thus, the aberrant expression of glial Cxs in MS lesions might impair neural functions in MS [5, 6]. Moreover, we found that the conditional knockout of oligodendroglial Cx47 exacerbated acute and chronic EAE [11], whereas the knockout of astroglial Cx30 attenuated chronic, but not acute, EAE [12]. These findings underscore the importance of glial Cxs in regulating neuroinflammation in addition to their metabolic functions [13–15]. Cx47 forms oligodendroglia-astroglia GJ channels with Cx43 whereas the loss of Cx47 leads to increased Cx43 hemichannels that secrete various bioactive molecules, including proinflammatory cytokines and chemokines [16, 17].

Interestingly, the in vivo imaging of activated astroglia by bioluminescence technology revealed that brain astroglia activated prior to the onset of EAE are detectable as early as 3 days post-immunization (dpi) and increase in number until the onset of acute EAE [18]. However, the role of brain astroglia activated by spinal cord inflammation before the onset of EAE is unknown. Therefore, we characterized the effects of brain astroglia on spinal cord inflammation in EAE. Here, we focused on Cx43 expressed on cortical astroglia because Cx43 has a critical role in controlling the CNS inflammatory milieu [17]. By using the glutamate aspartate transporter (GLAST)^+^ astroglia-specific inducible conditional knockout of *Cx43* in mice, which specifically ablates *Cx43* in brain gray matter astroglia [19], we demonstrated the remote proinflammatory effects of brain cortical astroglia on spinal cord inflammatory demyelination involving Cx43.

## Methods

### Ethical statement

The experimental procedures were designed to minimize the number of animals used and animal suffering. All animal experiments were carried out according to the guidelines for the proper conduct of animal experiments published by the Science Council of Japan, and ethical approval for the study was granted by the animal care and use committee of Kyushu University (#No. A25-196). The Animal Research: Reporting of In Vivo Experiments (ARRIVE) guidelines for animal research were followed.

### Animals and genotyping

*GLASTCreER*^*T2*^ mice on a C57BL/6 background were obtained from Dr. M. Götz (University of Ludwig-Maximilians) [19]. *Cx43* conditional “floxed” mice (*Cx43*^*fl/fl*^ mice) and Rosa26^*lacZ*^ mice on a C57BL/6 background were purchased from Jackson Laboratory (Bar Harbor, ME, USA). *GLASTCreER*^*T2*^ mice were crossed with *Cx43*^*fl/fl*^ mice or Rosa26^*lacZ*^ mice to generate *GLASTCreER*^*T2*^;*Cx43*^*fl/fl*^ mice or *GLASTCreER*^*T2*^;Rosa26^*lacZ*^ mice, respectively. DNA extracted from mouse ear-punched tissues was amplified by polymerase chain reaction (PCR) for genotyping using the following primers: GLAST F8 (5′-GAG GCA CTT GGC TAG GCT CTG AGG A-3′), GLAST R3 (5′-GAG GAG ATC CTG ACC GAT CAG TTG G-3′), and CreERT2 (5′-GGT GTA CGG TCA GTA AAT TGG ACA T-3′) were used for *GLASTCreER*^*T2*^ mice; *oIMR8086* (5′-CTT TGA CTC TGA TTA CAG AGC TTA A-3′) and *oIMR8087* (5′-GTC TCA CTG TTA CTT AAC AGC TTG A-3′) were used for *Cx43*^*fl/fl*^ mice; and *oIMR8545* (5′-AAA GTC GCT CTG AGT TGT TAT-3′), *oIMR8546* (5′-GGA GCG GGA GAA ATG GAT ATG-3′) and *oIMR8052* (5′-GCG AAG AGT TTG TCC TCA ACC-3′) were used for Rosa26^*lacZ*^ mice. The amplicons of (a) *GLASTCreER*^*T2*^ and wild-type (WT) alleles, (b) *Cx43* floxed and WT alleles, and (c) Rosa26^*lacZ*^ and WT alleles were (a) 410 and 720 bps, (b) 580 and 490 bps, and (c) 340 and 650 bps, respectively.

### Acute knockdown of *Cx43* in GLAST^+^ astroglia

**Fig. 1:**
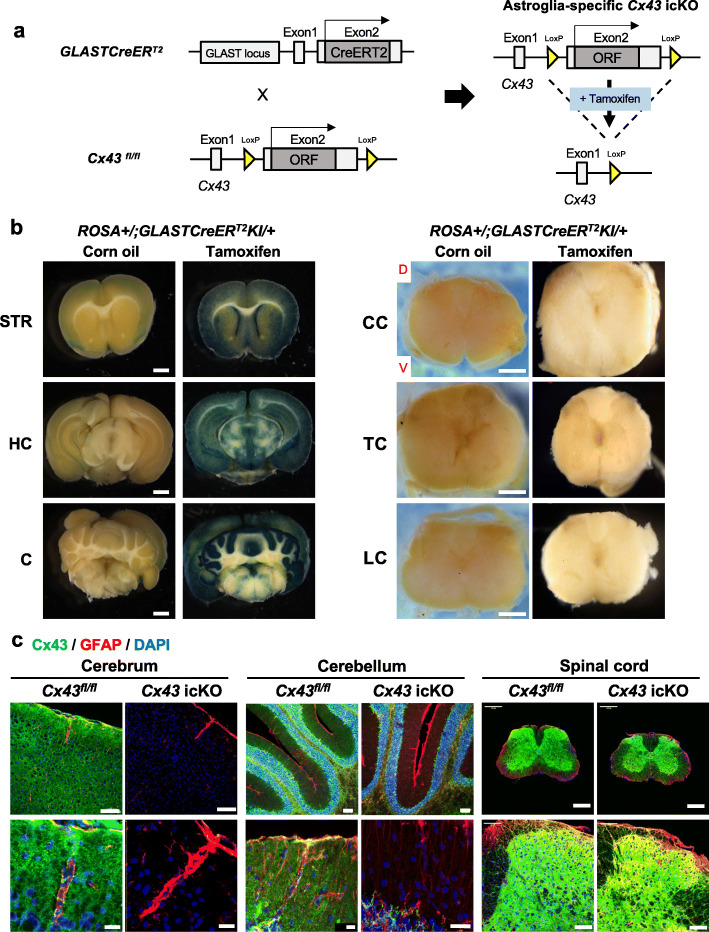
Strategy for astroglial *Cx43* icKO mouse generation. **a** A schematic diagram showing the generation of cortical astroglia-specific *Cx43* icKO mice. To obtain cortical astroglia-specific *Cx43* icKO mice, *GLASTCreER*^*T2*^ and *Cx43*^*fl/fl*^ mice (left) were bred to generate *GLASTCreER*^*T2*^;*Cx43*^*fl/fl*^ mice (right), which were injected with tamoxifen for recombination. **b** Evaluation of β-galactosidase-expressing cells after staining with 5-bromo-4-chloro-3-indolyl-β-galactopyranoside (X-gal), to identify CreER^T2^-mediated recombination. The X-gal^+^ regions (blue) represent areas with Cre-mediated recombination. Cre-mediated recombination occurred in the cerebral and cerebellar cortices, and the striatum, but little recombination occurred in the spinal cord. *STR* striatum, *HC* hippocampus, *C* cerebellum, *CC* cervical spinal cord, *TC* thoracic spinal cord, *LC* lumbar spinal cord, *D* dorsal, *V* ventral. Scale bars: 1 mm. **c** Double immunofluorescence for Cx43 (green) and GFAP (red). The expression of Cx43 was widely ablated in the cerebral and cerebellar cortices, but not in the spinal cord. GFAP expression in *Cx43* icKO mice was similar to that in *Cx43*^*fl/fl*^ mice. Each figure was counterstained with DAPI (blue). Scale bars: 100 μm for the upper row, 20 μm for the lower row

To induce the acute knockdown of *Cx43* specifically in gray matter astroglia, *GLASTCreER*^*T2*^;*Cx43*^*fl/fl*^ (*Cx43* icKO) mice were given an intraperitoneal injection of 1 mg of tamoxifen (Sigma-Aldrich, St. Louis, MO, USA) in 100 μl of corn oil twice per day for 5 consecutive days. We injected tamoxifen using the same dose and protocol for control mice (*Cx43*^*fl/fl*^) (Fig. 1 a). All mice were housed under a 12-h light/dark cycle and were allowed to acclimatize to conditions for at least 2 weeks before the administration of tamoxifen. All experiments were performed using 8–12-week-old female mice.

### β-galactosidase staining

*GLASTCreER*^*T2*^;Rosa26^*lacZ*^ mice were injected intraperitoneally with 1 mg of tamoxifen in 100 μl of corn oil twice per day for 5 consecutive days. Control *GLASTCreER*^*T2*^;Rosa26^*lacZ*^ mice were injected with corn oil only. To identify CreER^T2^-mediated recombination, we examined the floxed *LacZ* transgene by analyzing β-galactosidase-expressing cells after staining with 5-bromo-4-chloro-3-indolyl-β-galactopyranoside (X-gal) (Roche, Mannheim, Germany). Anesthetized mice were perfused with phosphate-buffered saline (PBS), and their brain and spinal cord were removed. The tissues were cleaned with PBS, moved to a 15-cm dish, and fixed in fresh 4% paraformaldehyde (PFA) for 1 h at 4 °C. Coronal brain and axial spinal cord sections from double-transgenic mice were incubated with 5% X-gal at 37 °C overnight, fixed in 4% PFA for 1 h at room temperature (RT) on a platform rocker, and washed with 70% ethanol until the tissues were bleached. *LacZ* expression was monitored by examining the appearance of blue spots in the tissue.

### Quantitative analysis of *Cx43* ablation in different CNS regions by Western blot

To confirm gray matter astroglia-specific *Cx43* ablation, we harvested brains and spinal cords of four *Cx43* icKO and four *Cx43*^*fl/fl*^ mice with tamoxifen treatment before the induction of EAE. Then, the cerebral cortices, cerebellar cortices, and spinal cords were dissected manually using a spatula. Tissues were lysed in ice-cold radioimmunoprecipitation assay buffer (50 mM Tris-HCl pH 7.0, 150 mM NaCl, 1% NP-40, 0.5% sodium deoxycholate, 0.1% sodium dodecyl sulfate) containing a protease inhibitor cocktail (#11836153001; Roche) using a BioMasher II (Fujifilm Wako, Osaka, Japan) and centrifuged at 10,000×*g* for 10 min at 4 °C. The protein concentrations were determined using a Pierce™ bicinchoninic acid (BCA) Protein Assay Kit (Thermo Fisher Scientific KK, Tokyo). The proteins were electrophoresed in 4–15% mini PROTEAN TGX precast gels (#4561086; Bio-Rad, Hercules, CA), transferred onto polyvinylidene fluoride membranes, and incubated at 4 °C overnight with a primary antibody against Cx43 (ab 11370, Abcam, Cambridge, UK). After washing, the membranes were incubated with 0.5% horseradish peroxidase-labeled IgG. The membrane-bound antibodies were detected with an enhanced chemiluminescence detection kit (ECL™ Western Blotting Detection Reagents; RPN2209; GE Healthcare, Chicago, IL) and analyzed with an image analyzer (ChemiDoc XRS System; Bio-Rad). After stripping the anti-Cx43 antibody, antibodies against β-actin (AC-15, Sigma, St. Louis, MO) were used to confirm equal sample loading.

### Induction and clinical evaluation of EAE

We induced EAE by immunization of mice with 4 mg/ml MOG_35–55_ peptide (TS-M704-P; MBL, Nagoya, Japan) emulsified in complete Freund’s adjuvant containing 1 mg/ml *Mycobacterium tuberculosis* H37RA (#231131; BD Difco, Lawrence, KS, USA) at a dose of 200 μg per mouse, followed by intraperitoneal injections of 300 ng pertussis toxin (# 180-A1; List Biological Laboratories Inc., Campbell, CA, USA) per mouse on days 0 and 2. Mice were examined daily for signs of EAE and scored as follows: 0, no disease; score 1, limp tail; 2, abnormal gait and hind limb weakness (shaking); 2.5, paralysis of one hind limb; 3, paralysis of two hind limbs; 3.5, ascending paralysis (able to move around); 4, tetraplegia; and 5, moribund (death).

### Tissue preparations

Animals were deeply anesthetized by isoflurane (Pfizer Japan Inc., Tokyo, Japan) and then perfused transcardially with PBS followed by 4% PFA in 0.1 M PBS. Spinal cords, brains, spleens, and inguinal lymph nodes were carefully dissected. The tissues were fixed overnight in cold 4% PFA at 4 °C then processed into paraffin sections (5 μm). For frozen sections, spinal cords, and brains were harvested and fixed overnight in 4% PFA using the same protocol as above and sequentially displaced with 20% sucrose and 30% sucrose in PBS solution for 24 h each at 4 °C. The resulting tissues were embedded in Tissue-Tek® optimal cutting temperature compound (4583, Sakura Finetek, Tokyo, Japan) and stored at − 80 °C.

### Histopathological and immunohistochemical analyses

Paraffin-embedded coronal brain, axial L4–5 spinal cord, spleen, and inguinal lymph node sections were subjected to hematoxylin and eosin (HE) and Klüver-Barrera (KB) staining, which is a combined Nissl and Luxol fast blue (LFB) stain for myelin. These sections were also examined by immunostaining using an indirect immunoperoxidase method. After deparaffinization, endogenous peroxidase was quenched with 0.3% hydrogen peroxide in absolute methanol for 30 min. The sections were permeabilized with 0.1% Triton in PBS (PBS-T) for 10 min, washed using Tris-HCl for 5 min, dipped into 10 mM citrate buffer, and then autoclaved (120 °C, 10 min). All sections were cooled to room temperature and incubated overnight at 4 °C with primary antibodies (Supplementary Table 1). The dilution solution comprised 5% normal goat serum and 1% BSA in 50 mM Tris-HCl (pH 7.6). After rinsing the slides the next day, sections were labeled with a streptavidin-biotin complex or an enhanced indirect immunoperoxidase method using Envision (K4003, Dako, Glostrup, Denmark). 3,3-Diaminobenzidine (DAB) tetrahydrochloride (D5637, Sigma-Aldrich, Tokyo, Japan) was used for DAB colored reactions. Finally, sections were counterstained with hematoxylin. Frozen sagittal sections of brains and spinal cords were cut on a Leica CM 1850 cryostat (Leica Microsystems GmbH, Wetzlar, Germany), incubated for 2 h at RT in blocking solution (PBS-T with 10% normal goat serum), and incubated at 4 °C with primary antibodies for 12 h. Detailed information of the primary antibodies used is provided in Supplementary Table 1. Next, the sections were incubated with secondary antibodies conjugated to Alexa Fluor 488 or 594 (1:1000; Thermo Fisher, Rockford, IL, USA) and 4,6-diamidino-2-phenylindole (DAPI) (Sigma-Aldrich, Tokyo, Japan), and mounted with PermaFluor (#TA-030-FM; Thermo Scientific, Fremont, CA). Images were captured using a confocal laser microscope system (Nikon A1; Nikon, Tokyo, Japan).

### Quantification of immunohistochemically stained images

Immunohistochemically stained sections of EAE samples were automatically scanned and quantified using ImageJ Analysis 1.51h software (https://imagej.nih.gov/ij/index.html). Briefly, for quantification, transverse sections of the spinal cord were divided by horizontal and vertical lines passing through the central canal (red-lines in Supplementary Fig. 1b), and the central gray matter area was manually excluded to define the white matter region of interest (the area encircled by a yellow line in Supplementary Fig. 1b). The positive-stained areas in this region were automatically measured and expressed as a percentage of the white matter area of interest. Quantifications were also performed on cerebral and cerebellar cortex, hippocampus, cerebellum, and L4–5 spinal cord sections immunostained with each antibody. These stained areas were also expressed as a percentage of the total region of interest (ROI). Quantification of the positive-stained areas in the hippocampal region was also performed by the optical dissection method [20]. Briefly, 100-μm^2^ ROIs in the stratum radiatum of the hippocampus were randomly set per section, and five sections from each mouse were used to calculate the mean positive-stained areas. The mean positive-stained areas were averaged in each mouse and then in each group.

### Microglial circularity analysis

ImageJ software was used to automatically calculate the circularity of microglial cells (circularity = 4πS/L2). Cells with circularity close to 1 were considered to have round morphology, which indicated an activated state [21].

### Isolation of splenocytes and draining lymph nodes

Spleens and inguinal lymph nodes were aseptically removed from pre-immunized phase mice, pre-EAE onset phase mice at dpi 10, and acute EAE phase mice at dpi 17 and dissociated into single cells as described previously [11]. Briefly, each spleen was squashed between two frosted glass slides and filtered through a 100-μm cell strainer. The cells were then centrifuged at 300×*g* for 10 min. After removal of the supernatant, PBS was added, and the cells were centrifuged as described above. Next, the cells were resuspended in red blood cell lysis buffer, incubated for 10 min, and centrifuged for 10 min at 300×*g*. We counted viable cells by using a hemocytometer and 0.4% Trypan blue staining.

### T cell proliferation assay

Around the peak of acute EAE, we collected mouse spleens. One hundred microliters of a splenocyte suspension (1 × 10^6^ cells/well) were placed in a 96-well plate, and 100 μl of Roswell Park Memorial Institute (RPMI) 1640 medium containing 1, 5, or 10 μg/ml MOG_35–55_ or 100 μl of RPMI 1640 medium (negative control) was added. The plates were incubated at 37 °C in a humidified atmosphere containing 5% CO_2_ for 72 h. For the last 18–24 h of incubation, 20 μl of 1 × bromodeoxyuridine (BrdU) solution was added to the wells. A background control without BrdU was included in the plate. After 72 h of incubation, T cell proliferation was assayed using a BrdU kit (ab126556; BrdU cell proliferation enzyme-linked immunosorbent assay (ELISA) Kit, Abcam) according to the manufacturer’s instructions. Briefly, the cells were fixed with 200 μl of fixing solution for 30 min and incubated with 100 μl of anti-BrdU monoclonal detector antibody for 1 h at RT. After washing, the cells were incubated with 100 μl of 1 × peroxidase-conjugated goat anti-mouse IgG filtered through a 0.22-μM syringe filter for 30 min at RT. The cells were then incubated with 100 μl of tetramethylbenzidine peroxidase in the dark for 30 min. After the addition of 100 μl of the stop reaction solution, the absorbances of the solutions in the wells were measured at wavelengths of 450 and 560 nm using a microplate reader (MTP-800AFC; Corona Electric, Hitachinaka, Japan).

### Multiplexed fluorescence immunoassay for cytokines in culture supernatants

To measure antigen-specific cytokine production, we aliquoted 100 μl of splenocyte suspension (1 × 10^6^ cells/well) into each well of a 96-well plate and cultured them for 72 h using the same protocol as above, except BrdU was not used. The concentrations of four cytokines/chemokines, interleukin (IL)-2, IL-17, interferon (IFN)-γ, and granulocyte-macrophage colony-stimulating factor (GM-CSF), in the supernatants were measured using a Bio-plex Pro™ Assay (M60-009RDPD; Bio-Rad) according to the manufacturer’s instructions. The cytokine/chemokine levels were calculated by referring to a standard curve for each molecule derived using various concentrations of standards assayed in the same manner as the supernatant samples. The detection limit for each molecule was determined by recovering the corresponding standard, and the lowest values with > 70% recovery were set as the lower detection limits. No samples were beyond the upper detection limits, and some samples were below the lower detection limits.

### Multiplexed fluorescence immunoassay for cytokines in the cerebrospinal fluid

To measure cytokine levels in mouse cerebrospinal fluid (CSF), we collected 3 μl of CSF from each mouse and used the LUNARIS™ mouse-12 plex cytokine kit (LMCY-10120S; AYOXXA Biosystems GmbH, Köln, Germany) according to the manufacturer’s protocol. We analyzed the concentrations of twelve cytokines/chemokines (IL-1β, IL-2, IL-6, tumor necrosis factor (TNF)-α, IFN-γ, IL-17a, IL-4, IL-5, IL-10, IL-13, IL-12p70, GM-CSF) in the CSF samples. The cytokine/chemokine levels were calculated by the multiplexed fluorescence bead-based immunoassay using the same protocol as above.

### Isolation of microglia

Mice in the pre-immunized and acute (dpi 17) phases of EAE were euthanized, and their spinal cords were isolated and collected in ice-cold 1× Hank’s Balanced Saline Solution (HBSS). Spinal cord and brain cells were isolated by a density-gradient technique, as previously described [11]. Briefly, spinal cord and brain tissues were minced with a tissue homogenizer to obtain a single-cell suspension. Stock isotonic Percoll® (10-fold dilution in 10× HBSS without Ca^2+^ and Mg^2+^) was added to the cell suspension to form a 30% Percoll gradient. A 70% Percoll gradient was pipetted underneath the 30% Percoll cell solution and centrifuged at 800×*g* for 40 min. Then, the myelin layer was removed and the mononuclear cell interphase was isolated and resuspended in 1× HBSS. After blocking with an anti-mouse cluster of differentiation (CD)16/32 monoclonal antibody (Sony Biotechnology, San Jose, CA) for 10–15 min on ice, the cells were stained with phycoerythrin (PE)-Cy7 anti-mouse CD11b (BD Pharmingen™, San Jose, CA), fluorescein isothiocyanate (FITC) anti-mouse CD45 (Invitrogen, Rockford, IL), and allophycocyanin (APC) anti-mouse Ly6C (BioLegend, San Diego, CA) monoclonal antibodies. The cells were then sorted and analyzed using an SH800 Cell Sorter (Sony Corporation, Tokyo, Japan) by gating on CD11b^+^CD45^int^Ly6C^−^ for microglial cells.

### Gene expression microarray

Total RNA was isolated from fresh whole cerebellar tissues or microglia isolated from the entire brain and spinal cord using an RNeasy Mini Kit (QIAGEN, Hilden, Germany) according to the manufacturer’s instructions. RNA samples were quantified with an ND-1000 spectrophotometer (NanoDrop™ Technologies, Wilmington, DE), and the quality was confirmed with a 2200 TapeStation (Agilent Technologies, Santa Clara, CA). Total RNA (2 ng) was amplified, labeled with a GeneChip® WT Pico Kit (Affymetrix, Santa Clara, CA), and hybridized to a GeneChip Mouse Transcriptome Array 1.0 (Affymetrix) according to the manufacturer’s instructions. All hybridized microarrays were scanned with an Affymetrix scanner (Affymetrix). Relative hybridization intensities and background hybridization values were calculated using Expression Console™ software (Affymetrix). The gene array results were uploaded to the gene expression omnibus repository (accession number: GSE148932) on the National Center for Biotechnology Information homepage (https://www.ncbi.nlm.nih.gov/geo/query/acc.cgi?acc=GSE148932).

### Differential expression analysis and filter criteria

The raw signal intensities of all samples were normalized by a quantile algorithm with Affymetrix Power Tool version 1.15.0 software (Affymetrix). To identify upregulated or downregulated genes, we calculated *Z*-scores and ratios (non-log-transformed fold changes) from the normalized signal intensities for each probe by comparisons between control and experimental samples. We then established criteria for differentially expressed genes (DEGs): upregulated genes had *Z*-scores ≥ 2.0 and ratios ≥ 1.5-fold, and downregulated genes had *Z*-scores ≤ − 2.0 and ratios ≤ 0.66. A gene-set enrichment analysis (GSEA) (www.broadinstitute.org/gsea) was performed to determine the enrichment score (ES), which indicated the degree to which each gene set was overrepresented at the top or bottom of a ranked list of genes. After the estimation of the statistical significance of the ES, we calculated the false discovery rate (FDR). When the normalized *P* value was < 0.05, and the FDR was < 0.25, the ES was considered significant. A heat map was generated using the “pheatmap” package (https://cran.rstudio.com/bin/windows/contrib/3.5/pheatmap_1.0.12.zip) or heatmap.2 (https://cran.r-project.org/web/packages/gplots/) in R software. Briefly, after log_2_ transformation of the original mRNA signal value, the distance from each gene median value (control) was calculated. The log_2_-transformed distance from each gene median value was represented as a color gradient on the heat map. If the log_2_-transformed distance was greater than 2, the color was changed to that for 2. Similarly, if the log_2_-transformed distance was less than − 2, the color was changed to that for − 2. Subsequently, the numbers − 2 to 2 were placed beside the color bar for the heat map to indicate the log_2_-transformed distances, which reflected fold differences in gene expression from less than 1/4 to more than 4, respectively.

### RNA extraction and RT-PCR

Total RNA was extracted from isolated microglia using an RNeasy Mini Kit (#74104; Qiagen), and cDNA was synthesized using ReverTra Ace qPCR RT Master Mix with gDNA Remover (#FSQ-301; Toyobo, Osaka, Japan). To measure low levels of RNA obtained from sorted cells, Perfecta Preamp Supermix (Quanta Biosciences, Beverly, USA) was used according to the manufacturer’s instructions. Real-time PCR was performed using the TaqMan Gene Expression Assay Protocol in the 7500 Real-Time PCR System (Applied Biosystems, Foster City, CA). Genes of interest were compared with and expressed as ratios relative to the reference gene (18S ribosomal RNA, *Rn18s*). Data were analyzed according to the Pfaffl method [22].

### Data analysis and statistics

All statistical analyses were blinded to the genotypes. All mice and samples were included in the analyses. Cell percentages and histological data for randomly selected L4–5 spinal cord sections were assessed. Each category included at least five lumbar spinal cord sections per mouse and ≥ 4 mice per condition. All data are presented as the mean ± standard error of the mean (SEM). The area under the curve (AUC) of overall disease severity was calculated for each mouse to compare the disease courses in *Cx43*^*fl/fl*^ and *Cx43* icKO mice using the non-parametric Mann–Whitney *U* test. The acute (days 7–18) and chronic (days 23–50) phases of EAE were assessed separately. The statistical significance of differences between data was determined by an unpaired *t* test, Mann–Whitney *U* test, or one-way analysis of variance (ANOVA). Values of *P* < 0.05 were considered statistically significant. All statistical analyses were performed using Graph Pad Prism 8.0 software (Graph Pad, La Jolla, CA). Gross differences between genotypes in the cytokine levels of mice with EAE in the onset phase were calculated by two-way ANOVA followed by Sidak’s multiple comparison tests. Heatmap and clustering analysis were generated using JMP Pro software (ver. 14.2.0; SAS Institute, Cary, NC) to detect cytokines with the highest degree of association to discriminate between Cx43^*fl/fl*^ and Cx43 icKO. Briefly, a two-way hierarchical cluster analysis of cytokines and each mouse genotype was performed, and the results are presented by dendrogram and heatmap according to the relative expression level of each cytokine.

## Results

### *Cx43* icKO in GLAST^+^ astroglia ablates Cx43 expression in brain gray matter but not in spinal cord

To investigate the *CreER*^*T2*^-mediated recombination in *GLASTCreER*^*T2*^;*Cx43*^*fl/fl*^ mice, we crossed *GLASTCreER*^*T2*^ mice with Rosa26^*lacZ*^ reporter mice (the genetic schema is shown in Fig. 1 a). We injected tamoxifen into these mice to induce *CreER*^*T2*^-mediated recombination. X-gal^+^ cells were abundant in the cerebral and cerebellar cortices and striatum. However, in the spinal cord, few positive cells were present in the dorsal horns (Fig. 1 b). We generated *GLASTCreER*^*T2*^;*Cx43*^*fl/fl*^ mice to achieve the inducible conditional ablation of *Cx43* in the brain gray matter, wherein Cx43 is ablated from GLAST^+^ astroglia after tamoxifen injection. Tamoxifen-treated *GLASTCreER*^*T2*^;*Cx43*^*fl/fl*^ mice are designated as *Cx43* icKO mice hereafter. Immunohistochemical analysis showed that Cx43 was widely deleted in the cerebral and cerebellar cortices and striatum, whereas Cx43 expression in the spinal cord was unchanged in *Cx43* icKO mice compared with corn oil-injected control mice (Fig. 1 c). Western blot analysis showed a significant decrease of Cx43 protein levels in the cerebral and cerebellar cortices (*P* = 0.0434, and *P* = 0.0454, respectively), but not in the spinal cord (*P* = 0.6833) (Supplementary Fig. 2). This finding is consistent with the finding that GLAST expression is successively decreased from neonates to adults, in whom detectable GLAST signals in the spinal cord are only present in the dorsal horns and around the central canal [23]. These results indicate that *Cx43* icKO in GLAST^+^ astroglia induces the specific loss of astroglial Cx43 in the brain gray matter, particularly in the cerebral and cerebellar cortices, but not in the spinal cord. In addition, no pathology was found in either brain or spinal cord in the pre-immunized phase in *Cx43* icKO mice compared with *Cx43*^*fl/fl*^ mice by HE staining or by MBP^+^, LFB^+^, and Iba1^+^ area % (Supplementary Fig. 1).

### Brain gray matter astroglia-specific *Cx43* ablation attenuates acute and chronic EAE clinical signs

**Fig. 2:**
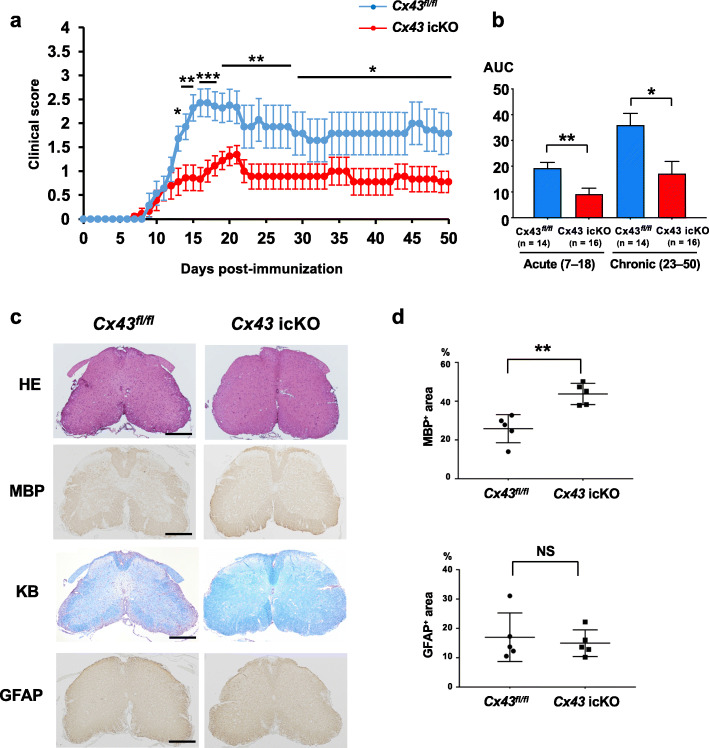
Comparison of EAE between *Cx43* icKO and *Cx43*^*fl/fl*^ mice. **a** Clinical scores of EAE (means ± SEM) over time in *Cx43* icKO (red, *n* = 16) and *Cx43*^*fl/fl*^ mice (blue, *n* = 14). Horizontal bars in the graph show the periods with significant differences between the two genotypes. *P < 0.05, **P < 0.01, ***P < 0.001. **b** The AUC of clinical scores in the acute (dpi 7–18) and chronic (dpi 23–50) phases. *P < 0.05, **P < 0.01. **c** Immunohistochemical staining of the lumbar spinal cord from mice of each genotype at dpi 17. *Cx43* icKO mice had fewer infiltrated inflammatory cells by HE staining (top row) and smaller demyelinated areas as shown by anti-MBP immunostaining and KB staining (second and third rows) than *Cx43*^*fl/fl*^ mice. Anti-GFAP immunostaining showed no significant differences between the two genotypes (bottom row). Scale bars: 200 μm. **d** Quantitative analysis showed significant differences in MBP^+^ areas (top), but not GFAP^+^ areas (bottom), between the two genotypes. **P < 0.01. *NS* not significant

Mice of both genotypes showed typical EAE manifestations, such as paraparesis and quadriparesis, but no atypical signs, such as ataxic gait and hemiparesis, and the incidence rate did not differ significantly between the two genotypes [*Cx43* icKO vs. *Cx43*^*fl/fl*^ = 88.9% (16/18) vs. 100% (14/14)] (Table 1). However, clinical signs of EAE were significantly milder in *Cx43* icKO mice than in *Cx43*^*fl/fl*^ mice from acute to chronic phases [AUC of clinical scores, acute phase: *Cx43* icKO vs. *Cx43*^*fl/fl*^ = 9.2 ± 2.3 vs. 19.3 ± 2.2, *P* = 0.0046, and chronic phase: *Cx43* icKO vs. *Cx43*^*fl/fl*^ = 17.1 ± 4.8 vs. 35.9 ± 4.6, *P* = 0.0149] (Fig. 2 a, b), Moreover, the clinical scores were significantly lower in *Cx43* icKO mice than in *Cx43*^*fl/fl*^ mice from dpi 13 to 50 including the peak score [*Cx43* icKO vs. *Cx43*^*fl/fl*^ = 1.6 ± 0.2 vs. 2.7 ± 0.2, *P* = 0.0008]. The onset was also delayed in *Cx43* icKO mice, but the difference did not reach statistical significance compared with *Cx43*^*fl/fl*^ mice [*Cx43* icKO vs. *Cx43*^*fl/fl*^ = 14.6 ± 1.0 vs. 12.4 ± 0.6, *P* = 0.0884] (Table 1).

**Table 1:** Comparison of clinical signs of EAE between *Cx43* icKO and *Cx43*^*fl/fl*^ mice

Genotype	Incidence	Onset (dpi)	Peak clinical scores	AUC*	
				acute	chronic
*Cx43* icKO	88.9% (16/18)	14.6 ± 1.0	1.6 ± 0.2	9.2 ± 2.3	17.1 ± 4.8
*Cx43* fl/fl	100% (14/14)	12.4 ± 0.6	2.7 ± 0.2	19.3 ± 2.2	35.9 ± 4.6
*P value*	NS	NS	0.0008	0.0046	0.0149

*Area under the curve (AUC) was calculated for dpi 7–18 and 23–50 in the acute and chronic phases, respectively

*Cx* connexin, *dpi* days post-immunization, *fl* flox, *icKO* inducible conditional knock-out, *NS* not significant

### Brain gray matter astroglia-specific *Cx43* ablation attenuates inflammatory demyelination in the spinal cord

KB staining showed significant decrease of LFB^+^ myelinated areas in the lumbar spinal cord at dpi 17 compared with the pre-immunized phase in mice of both genotypes [the pre-immunized phase vs. dpi 17 = 90.25 ± 0.85 vs. 55.0 ± 2.45, *P* < 0.0001 in *Cx43*^*fl/f*l^ mice, and 89.50 ± 1.76 vs. 74.0 ± 2.86, *P* = 0.0012 in *Cx43* icKO mice]; however, there were significantly more LFB^+^ myelinated areas in *Cx43* icKO mice than in *Cx43*^*fl/fl*^ mice at dpi 17 (*P* = 0.0002) (Fig. 2 b, c, Supplementary Fig. 1b, c). Furthermore, MBP^+^ myelinated areas in the lumbar spinal cord were significantly decreased at dpi 17 compared with the pre-immunized phase in mice of both genotypes [the pre-immunized phase vs. dpi 17 = 76.40 ± 1.77 vs. 48.78 ± 2.26, *P* < 0.0001 in *Cx43*^*fl/f*l^ mice, and 74.15 ± 1.79 vs. 60.38 ± 2.13, *P* = 0.0024 in *Cx43* icKO mice], whereas they were significantly larger in *Cx43* icKO mice compared with *Cx43*^*fl/fl*^ mice at dpi 17 (*P* = 0.012) (Fig. 2 c, d, Supplementary Fig. 1d, ed, e). In contrast, glial fibrillary acidic protein (GFAP)^+^ astroglial areas were similar between the two genotypes [*Cx43* icKO vs. *Cx43*^*fl/fl*^ (%) = 15.0 ± 4.6 vs. 17.0 ± 8.3, *P* = 0.83] (Fig. 2 c, d). In the hippocampus, no difference was observed in either MBP^+^ or LFB^+^ area % between the two genotypes even at dpi 17 (Supplementary Fig. 1a–d).

**Fig. 3:**
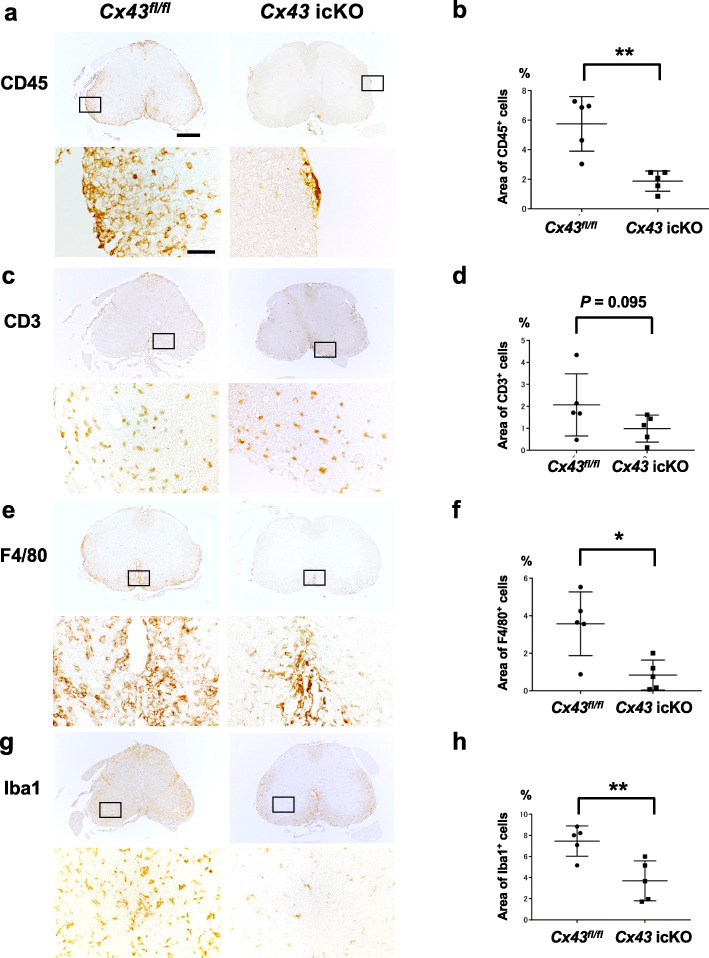
Comparison of inflammatory cell infiltrates between *Cx43* icKO and *Cx43*^*fl/fl*^ mice during acute EAE. Lumbar spinal cord samples from mice of each genotype in the acute phase (dpi 17) of EAE were immunostained by the indicated antibody (**a**, **c**, **e**, **g**) and immunostained cell densities were quantified (**b**, **d**, **f**, **h**). Significantly fewer CD45^+^ (**a**, **b**), F4/80^+^ (**e**, **f**), and Iba1^+^ (**g**, **h**) cells infiltrated into the lumbar spinal cords of *Cx43* icKO mice than *Cx43*^*fl/fl*^ mice whereas CD3^+^ T cell infiltration tended to be deceased in *Cx43* icKO mice compared with *Cx43*^*fl/fl*^ mice (**c**, **d**). The rectangle in the upper row indicates the area enlarged in the lower row. Scale bar for whole spinal cord image: 200 μm, scale bar for enlarged image: 20 μm. *P < 0.05, **P < 0.01

Regarding immunocytes, fewer infiltrating CD45^+^ cells in the lumbar spinal cord were observed in *Cx43* icKO mice compared with *Cx43*^*fl/fl*^ mice [*Cx43* icKO vs. *Cx43*^*fl/fl*^ (%) = 1.88 ± 0.31 vs. 5.75 ± 0.83, *P* = 0.0023] (Fig. 3 a, b). Numbers of CD3^+^ T cells were lower in *Cx43* icKO mice than in *Cx43*^*fl/fl*^ mice in the lumbar spinal cord, although the difference was not statistically significant [*Cx43* icKO vs. *Cx43*^*fl/fl*^ (%) = 0.99 ± 0.27 vs. 2.07 ± 0.63, *P* = 0.095] (Fig. 3 c, d). However, significantly fewer F4/80^+^ activated macrophages and Iba1^+^ microglia were present in *Cx43* icKO mice compared with *Cx43*^*fl/fl*^ mice in the lumbar spinal cord [F4/80: *Cx43* icKO vs. *Cx43*^*fl/fl*^ (%) = 0.83 ± 0.36 vs. 3.56 ± 0.77, *P* = 0.0122; Iba1: 3.70 ± 0.85 vs 7.47 ± 0.64, *P* = 0.0075] (Fig. 3 e–h). As a result, numbers of Iba1^+^ microglia were significantly increased at dpi 17 compared with the pre-immunized phase in *Cx43*^*fl/fl*^ mice (*P* < 0.0001), whereas they were not significantly different between the pre-immunized phase and dpi 17 in *Cx43* icKO mice (Supplementary Fig. 1f, g). In the hippocampus, the Iba1^+^ area was minimally increased in both genotypes at dpi 17 compared with the pre-immunized phase [the pre-immunized phase vs. dpi 17 = 0.0260 ± 0.0033 vs. 0.2175 ± 0.0375, *P* = 0.0002 in *Cx43*^*fl/f*l^ mice, and 0.0285 ± 0.0047 vs. 0.2075 ± 0.0202, *P* = 0.0004 in *Cx43* icKO mice] while there was no significant difference in Iba1^+^ area % between both genotypes at dpi 17. No obvious infiltration of peripheral immunocytes was found in the hippocampus by HE staining and CD3 immunostaining [*Cx43* icKO vs. *Cx43*^*fl/fl*^ (%) = 0.295 ± 0.071 vs. 0.390 ± 0.104, *P* = 0.813] even at dpi 17 (Supplementary Fig. 1e, f).

### MOG-specific T cell responses are unaltered in *Cx43* icKO mice

Because Cx43 is also expressed by peripheral immunocytes, such as T cells [24, 25], we immunostained for Cx43 in the spleen and the draining inguinal lymph nodes. *Cx43* icKO and *Cx43*^*fl/fl*^ mice showed similar expression patterns of Cx43 in both tissues (Supplementary Fig. 3a). Furthermore, splenocytes isolated from *Cx43* icKO and *Cx43*^*fl/fl*^ mice in the acute phase (dpi 17) showed no significant differences in MOG_35–55_-specific proliferation (Supplementary Fig. 3b). The levels of proinflammatory cytokines such as IL-2, IL-17, IFN-γ, and GM-CSF secreted in culture media were similarly increased in both mouse genotypes in response to escalating concentrations of MOG_35–55_ (Supplementary Fig. 3c–f).

### Brain gray matter astroglia-specific *Cx43* ablation enhances A2 astroglial gene expression in the pre-immunized phase and attenuates A1 astroglial gene up-regulation at the onset of EAE

**Fig. 4:**
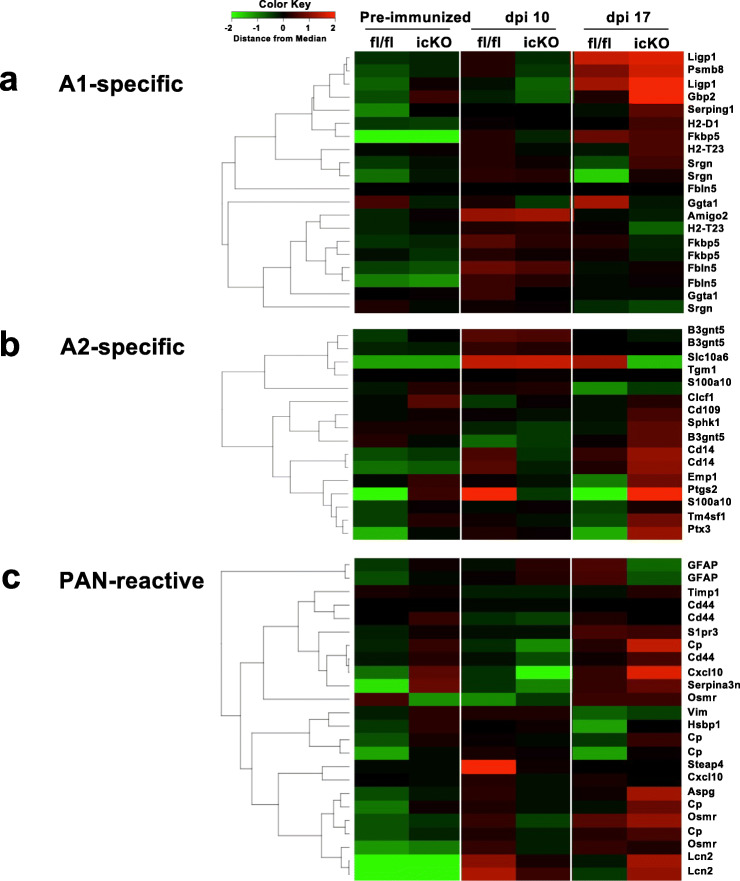
Gene expression microarray data for *Cx43* icKO and *Cx43*^*fl/fl*^ mouse brains during acute EAE. Heat maps comparing the expression of A1-specific (**a**), A2-specific (**b**), and pan-reactive (**c**) gene transcripts in RNA samples from the brains of *Cx43* icKO and *Cx43*^*fl/fl*^ mice (*n* = 3 per group) in the pre-immunized, onset (dpi 10), and peak (dpi 17) phases of EAE. To create the heat maps, the original mRNA signal values were subjected to log2 transformation, and the distance from the median value (control) of each gene was calculated. The numbers − 2 to 2 beside the color bar indicate log2-transformed fold changes, reflecting fold differences in gene expression from less than 1/4 to more than 4, respectively

First, we compared the temporal changes in the expression of astroglia-related gene clusters using whole cerebellar tissues from *Cx43* icKO and *Cx43*^*fl/fl*^ mice by GSEA. The cerebellum was used because Cx43 loss was most remarkable in the cerebellar cortex and there is less non-cortical tissue in the cerebellum than in the cerebrum. In *Cx43*^*fl/fl*^, the expression levels of A1-specific (proinflammatory gene cluster induced by neuroinflammation), A2-specific (anti-inflammatory gene cluster induced by ischemia), and pan-reactive (gene cluster induced by neuroinflammation *and* ischemia) genes were significantly higher in the onset phase than in the pre-immunized phase (normalized *P* values for A1-specific, A2-specific, and pan-reactive astroglial gene signatures: 0.038, 0.011, and 0.0016, respectively) (Fig. 4 a–c, Table 2). When the pre-immunized and peak phases were compared, the expression levels of A1- and pan-reactive gene sets, but not the A2-specific gene set, were still significantly greater in the peak phase than in the pre-immunized phase (normalized *P* values for A1-specific, A2-specific, and pan-reactive astroglial gene signatures: 0.004, 0.284, and 0.008, respectively). Finally, a comparison of gene expression between the onset and peak phases showed that the expression levels of A2-specific gene sets, but not of A1-specific or pan-reactive gene sets, were significantly greater in the peak phase than in the onset phase (normalized *P* values for A1-specific, A2-specific, and pan-reactive astroglial gene signatures: 0.885, 0.024, and 0.225, respectively).

**Table 2:** Comparison of astroglial gene expression among the pre-immunized, onset, and peak phases of EAE in *Cx43* icKO and *Cx43*^*fl/fl*^ mice

Gene cluster	Pre vs. Onset		Pre vs. Peak		Onset vs. Peak	
	*Cx43* fl/fl	*Cx43* icKO	Cx43 fl/fl	*Cx43* icKO	*Cx43* fl/fl	*Cx43* icKO
A1 (Pro-inflammatory)	0.038* (Onset)	NS	0.004 (Peak)	< 0.001 (Peak)	NS	NS
A2 (Anti-inflammatory)	0.011 (Onset)	NS	NS	0.010 (Peak)	0.024 (Onset)	NS
Pan-reactive	0.0016 (Onset)	NS	0.008 (Peak)	0.057 (Peak)	NS	0.025 (Peak)

A disease phase showing significantly higher levels of the relevant gene expression than their comparators is indicated in parentheses. Onset is dpi 10 and Peak is dpi 17

*Cx* connexin, *dpi* days post-immunization, *EAE* experimental autoimmune encephalomyelitis, *ES* enrichment score, *fl* flox, *icKO* inducible conditional knock-out, *NS* not significant, *Pre* pre-immunized

*P values of the ES plots are shown

In *Cx43* icKO, the expression levels of A1-specific, A2-specific, and pan-reactive genes were unchanged between the onset and pre-immunized phases (normalized *P* values for A1-specific, A2-specific, and pan-reactive astroglial gene signatures: 0.684, 0.556, and 0.392, respectively). A comparison between the pre-immunized and peak phases showed that A1-specific, A2-specific, and pan-reactive gene sets were all upregulated in the peak phase compared with the pre-immunized phase (normalized *P* values for A1-specific, A2-specific, and pan-reactive astroglial gene signatures: < 0.001, 0.010, and 0.057, respectively). Finally, a comparison of the gene expression changes between onset and peak phases revealed that pan-reactive gene sets were significantly upregulated in the peak phase compared with the onset phase but A1-specific and A2-specific gene sets were unchanged between the onset and peak phases (normalized *P* values for A1-specific, A2-specific, and pan-reactive astroglial gene signatures: 0.093, 0.122, and 0.025, respectively). These findings suggest that the increase in the A1-specific and pan-reactive gene sets in the onset phase was attenuated in *Cx43* icKO mice compared with *Cx43*^*fl/fl*^ mice.

To precisely elucidate the effects of *Cx43* ablation on A1-specific, A2-specific, and pan-reactive gene expression, we performed GSEA analysis between *Cx43*^*fl/fl*^ and *Cx43* icKO mice. In the pre-immunized phase, an upregulation of pan-reactive gene sets and especially the A2-specific gene sets was observed in *Cx43* icKO mice compared with *Cx43*^*fl/fl*^ mice (normalized *P* values for A1-specific, A2-specific, and pan-reactive astroglial gene signatures: 0.208, < 0.001, and 0.0018, respectively) (Fig. 4 a–c, Table 3). In the onset phase, A1-specific and pan-reactive gene expression was significantly greater in *Cx43*^*fl/fl*^ mice than in *Cx43* icKO mice (normalized *P* values for A1-specific, A2-specific, and pan-reactive astroglial gene signatures: 0.018, 0.068, and 0.0017, respectively). In the peak phase, no significant difference was found between the two mouse genotypes (normalized *P* values for A1-specific, A2-specific, and pan-reactive astroglial gene signatures: 0.308, 0.0546, and 0.159, respectively). These findings suggest that the marked upregulation of A2-specific gene sets in the pre-immunized phase is characteristic of *Cx43* icKO mice and might be associated with the attenuated A1-specific and pan-reactive gene expression in the onset phase in these mice.

**Table 3:** Comparison of astroglial gene expression between *Cx43*^*fl/fl*^ and *Cx43* icKO mice in the pre-immunized, onset, and peak phases of EAE

Gene cluster	*Cx43* fl/fl vs. *Cx43* icKO		
	Pre-immunized	Onset	Peak
A1 (Pro-inflammatory)	NS	0.018 (fl/fl)	NS
A2 (Anti-inflammatory)	< 0.001 (icKO)	NS	NS
Pan-reactive	0.0018 (icKO)	0.0017 (fl/fl)	NS

Mouse genotypes showing significantly higher levels of the relevant gene expression than their comparators are indicated in parentheses. Onset is dpi 10 and Peak is dpi 17

*Cx* connexin, *dpi* days post-immunization, *EAE* experimental autoimmune encephalomyelitis, *ES* enrichment score, *fl* flox, *icKO* inducible conditional knock-out

*P values of the ES plots are shown

### Brain gray matter astroglia-specific *Cx43* ablation reduces the upregulation of proinflammatory A1 astroglia markers in the peak phase of EAE

**Fig. 5:**
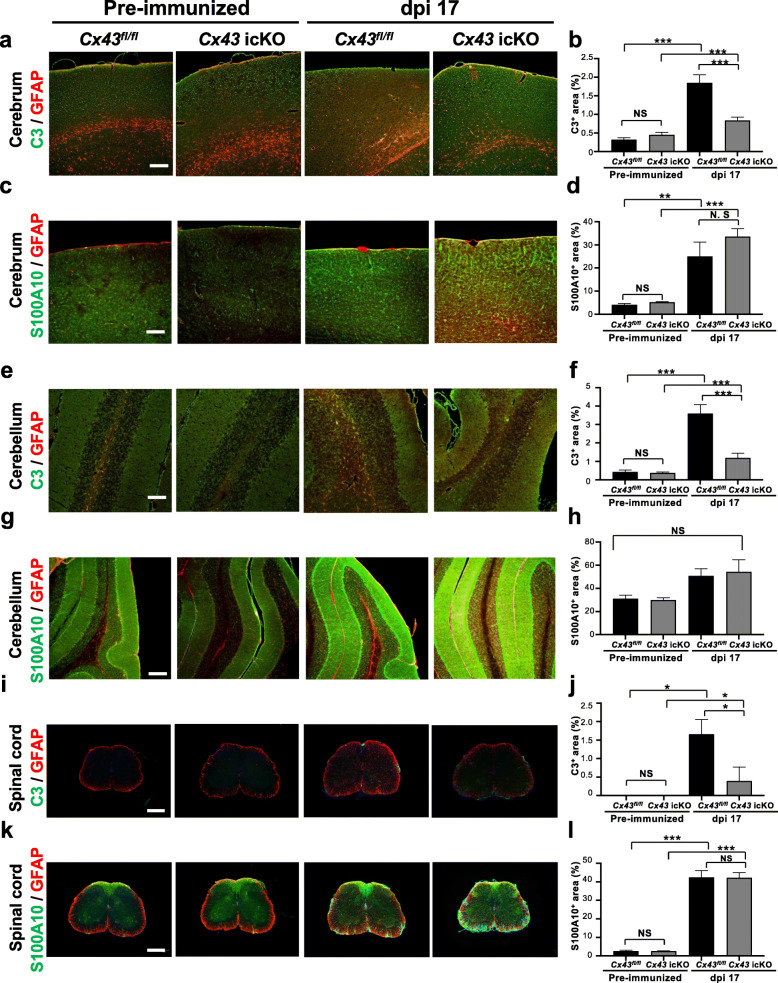
Immunohistochemistry of representative A1 and A2 astroglia markers in GFAP^+^ astroglia during acute EAE. Double immunofluorescence for C3 (green), an A1 astroglia marker, and GFAP (red) (**a**, **e**, **i**) or S100A10 (green), A2 astroglia markers, and GFAP (red) (**c**, **g**, **k**) in the cerebrum (**a**, **c**), the cerebellum (**e**, **g**), and the spinal cord (**i**, **k**) from *Cx43*^*fl/fl*^ and *Cx43* icKO mice in the pre-immunized and peak phases (dpi 17) of EAE. Quantitative analysis results of C3/GFAP double-positive (**b**, **f**, **j**) and S100A10/GFAP double-positive areas (**d**, **h**, **l**) in each region of the CNS from *Cx43* icKO and *Cx43*^*fl/fl*^ mice in the pre-immunized and peak phases (dpi 17) of EAE are shown. All data are presented as the means ± SEM of 4 mice per group. The statistical significance of differences in each comparison was analyzed by one-way ANOVA, followed by Tukey’s post-hoc analysis. All figures were counterstained by DAPI (blue). *P < 0.05, **P < 0.01, ***P < 0.001. *NS* not significant. Scale bars: 500 μm (**i**, **k**), 200 μm (**a**, **g**), and 100 μm (**c**, **e**)

We immunohistochemically examined the expression of representative A1 and A2 astroglia markers in the cerebrum, cerebellum, and spinal cord lesions in the pre-immunized and peak (dpi 17) phases, based on a recent report [26]. We found a significant decrease in GFAP^+^ astroglia expressing C3, a representative A1 marker, in the cerebrum, cerebellum, and lumbar spinal cord of *Cx43* icKO mice compared with *Cx43*^*fl/fl*^ mice in the peak phase (*P* = 0.0005, 0.0003, and 0.0435, respectively) (Fig. 5 a, b, e, f, i, j), whereas no significant change in GFAP^+^ astroglia expressing S100A10, a representative A2 marker, was observed between the two mouse genotypes (Fig. 5 c, d, g, h, k, l). These findings suggest that brain gray matter astroglia-specific *Cx43* ablation suppresses astroglial activation toward a proinflammatory phenotype during acute EAE.

### Brain gray matter astroglia-specific *Cx43* ablation promotes anti-inflammatory spinal cord microglia

**Fig. 6:**
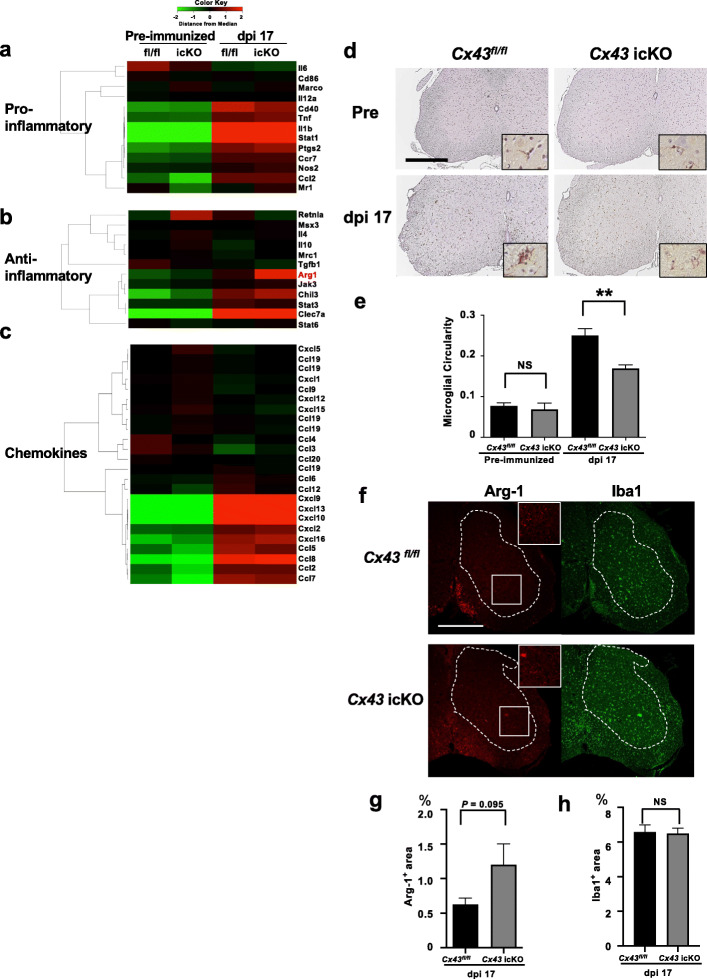
Microarray analysis and immunohistochemistry of *Cx43* icKO and *Cx43*^*fl/fl*^ microglia isolated from whole spinal cord and brain tissues during acute EAE. **a**–**c** Heat maps comparing the expression of proinflammatory (**a**), anti-inflammatory (**b**), and chemokine-related (**c**) gene transcripts in RNA samples from microglia isolated from the spinal cords of *Cx43* icKO and *Cx43*^*fl/fl*^ mice (*n* = 3 per group) in the pre-immunized and peak phases (dpi 17) of EAE. **d** Immunostaining of lumbar spinal cord lesions by anti-Iba1 antibody in the pre-immunized and peak phases (dpi 17) of EAE. Insets indicate enlarged images in each figure. Scale bar: 200 μm. **e** Quantification of microglia circularity in the spinal cord in the preimmunized and peak (dpi 17) phases of EAE. The statistical significance of differences for each comparison was analyzed by one-way ANOVA, followed by Tukey’s post-hoc analysis. *n* = 4–5 for each group. ** *P* < 0.01, NS = not significant. **f** Immunofluorescence of lumbar spinal cord lesions in the peak (dpi 17) phase of EAE. The red color indicates i immunolabeling by anti-arginase-1 antibody, and the green color indicates immunolabeling by anti-Iba1 antibody. The dotted line on each figure indicates the boundary between the gray and white matter of the spinal cord. Insets indicate enlarged images in each figure. Scale bar: 200 μm. **g** Quantification of the Arg-1^+^ area fraction (%) in the spinal cord gray matter in the peak phase of EAE. **h** Quantification of the Iba1^+^ area fraction (%) in the spinal cord gray matter in the peak phase of EAE. Student’s *t* test was performed for statistical analysis. *n* = 5 for each genotype. NS = not significant

To analyze the effects of cortical astroglia-specific *Cx43* ablation on microglia, we compared the temporal changes in the expression levels of pro- and anti-inflammatory genes in microglia isolated from the whole spinal cords of *Cx43* icKO and *Cx43*^*fl/fl*^ mice between the pre-immunized and peak (dpi 17) phases by GSEA. First, in *Cx43*^*fl/fl*^ mice, pro- and anti-inflammatory gene expression levels in microglia were significantly higher in the peak phase than in the pre-immunized phase (normalized *P* values for pro-, and anti-inflammatory gene signatures: < 0.001, and 0.0115, respectively) (Fig. 6 a, b, Table 4). In *Cx43* icKO mice, pro-, but not anti-inflammatory gene expression levels, were significantly higher in the peak phase than in the pre-immunized phase (normalized *P* values for pro-, and anti-inflammatory gene signatures: < 0.001, and 0.0748, respectively).

**Table 4:** Comparison of gene expression in microglia isolated from the spinal cords of *Cx43* icKO and *Cx43*^*fl/fl*^ mice between the pre-immunized and peak phases of EAE

	Pre-immunized vs. Peak	
Gene cluster	*Cx43* fl/fl	*Cx43* icKO
Pro-inflammatory	< 0.001 (Peak)	< 0.001 (Peak)
Anti-inflammatory	0.0115 (Peak)	NS
Chemokines	< 0.001 (Peak)	< 0.001 (Peak)

The disease phases showing significantly higher levels of the relevant gene expression than their comparators are indicated in parentheses. Peak is dpi 17

*Cx* connexin, *dpi* days post-immunization, *EAE* experimental autoimmune encephalomyelitis, *ES* enrichment score, *fl* flox, *icKO* inducible conditional knock-out, NS = not significant

*P values of the ES plots are shown.

Next, we compared the changes in microglial pro- and anti-inflammatory gene expression levels between *Cx43* icKO and *Cx43*^*fl/fl*^ mice during the EAE disease course by GSEA. In the pre-immunized phase, the expression levels of anti-inflammatory genes were significantly higher and those of proinflammatory genes were lower in *Cx43* icKO mice than in *Cx43*^*fl/fl*^ mice (normalized *P* values for pro-, and anti-inflammatory gene signatures: 0.0249, and 0.0307, respectively) (Fig. 6 a, b, Table 5). Conversely, no differences in pro- or anti-inflammatory gene expression levels were detected between the two genotypes of mice in the peak phase (normalized *P* values for pro-, and anti-inflammatory gene signatures: 0.6143 and 0.3076, respectively). These findings suggest that brain gray matter astroglia-specific *Cx43* ablation promotes anti-inflammatory spinal cord microglia in the pre-immunized state.

**Table 5:** Comparison of gene expression between microglia isolated from the spinal cords of *Cx43*^*fl/fl*^ and *Cx43* icKO mice in the pre-immunized and peak phases of EAE

	*Cx43* fl/fl vs. *Cx43* icKO	
Gene cluster	Pre-immunized	Peak
Pro-inflammatory	0.0249 (fl/fl)	NS
Anti-inflammatory	0.0307 (icKO)	NS
Chemokines	0.017 (fl/fl)	NS

Mouse genotypes showing significantly higher levels of the relevant gene expression than their comparators are indicated in parentheses. Peak is dpi 17

*Cx* connexin, *dpi* days post-immunization, *EAE* experimental autoimmune encephalomyelitis, *ES* enrichment score, *fl* flox, *icKO* inducible conditional knock-out, *NS* not significant

*P values of the ES plots are shown

### Brain gray matter astroglia-specific *Cx43* ablation suppresses chemokine-related gene expression in spinal cord microglia

To clarify the differences in chemoattractant production between the two genotypes of microglia, we performed GSEA analysis for chemokine gene signatures. As expected, chemokine-related genes were significantly upregulated in both genotypes in the peak phase of EAE compared with the pre-immunized phase (normalized *P* < 0.001 in both genotypes) (Fig. 6 c, Table 4). The overall chemokine-related gene expression was significantly greater in *Cx43*^*fl/fl*^ microglia than in *Cx43* icKO microglia in the pre-immunized phase, although no significant difference was observed between the two genotypes in the peak phase (normalized *P* values for chemokine-related gene signatures in the pre-immunized phase and peak phase of EAE: 0.017 and 0.919, respectively) (Fig. 6 c, Table 5). In particular, *Cx43* icKO spinal microglia had significantly fewer mRNA signals for *CC chemokine ligand* (*Ccl*)*2*, *Ccl5*, *Ccl7*, and *Ccl8* in the pre-immunized phase (*Z-*scores: − 2.29, − 2.33, − 3.80, and − 3.37, respectively) and for *CXC chemokine ligand* (*Cxcl9*) in the peak phase (*Z-*score: − 1.55) compared with *Cx43*^*fl/fl*^ mice. By real-time RT-PCR analysis, reduced expression of *Ccl2* and *Ccl7* in the pre-immunized phase (78.52% and 66.01% decrease, respectively) and reduced expression of *Ccl5* and *Ccl2* at the peak of acute EAE (47.12% and 21.03% decrease, respectively) were found in *Cx43* icKO mice compared with *Cx43*^*fl/fl*^ mice, although the changes were not evident for *Ccl8* (Supplementary Fig. 4). These findings suggest that brain gray matter astroglia-specific *Cx43* ablation suppresses chemokine-related gene expression in spinal cord microglia, particularly in the pre-immunized phase.

### Reduced activation status and enhanced anti-inflammatory phenotype of spinal microglia in *Cx43* icKO mice

The activation state of microglia in the spinal cord lesions was examined morphologically and immunohistochemically in both genotypes of mice in the pre-immunized and peak phases of EAE. The circularity of microglia in the spinal cord lesions was significantly lower in *Cx43* icKO mice than in *Cx43*^*fl/fl*^ mice in the peak phase (*P* = 0.0018) (Fig. 6 d, e). We then performed immunohistochemistry for arginase 1 (Arg-1), a representative anti-inflammatory marker, in the spinal cord lesions to verify the increased *Arg1* mRNA levels (Fig. 6 b). The Arg-1^+^ area fraction was quantified only in the spinal gray matter to avoid contamination by peripheral immunocytes that infiltrated mainly into the white matter. The Arg-1^+^ area fraction in the gray matter of the lumbar spinal cord tended to be increased in *Cx43* icKO mice compared with *Cx43*^*fl/fl*^ mice (*P* = 0.0952), whereas there was no significant change in the Iba1^+^ area (Fig. 6 f, g, h). These observations are consistent with the GSEA results demonstrating a reduced activation status and enhanced anti-inflammatory phenotype of *Cx43* icKO microglia compared with *Cx43*^*fl/fl*^ microglia, even in the peak phase of EAE.

### Decreased CSF cytokine levels in *Cx43* icKO mice in the onset phase of EAE

**Fig. 7:**
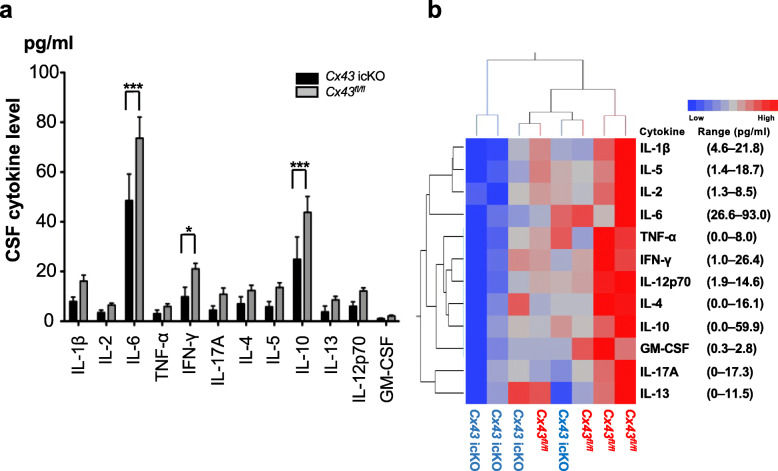
CSF cytokine levels in *Cx43* icKO and *Cx43*^*fl/fl*^ mice in the onset phase of EAE. **a** Mouse CSF was withdrawn from the foramen magna in the onset phase (dpi 10) of EAE and analyzed by a multiplexed fluorescence immunoassay. Each bar represents the mean cytokine level ± SEM (*n* = 3). Data were statistically analyzed by two-way ANOVA, followed by Sidak’s multiple comparison tests. *P < 0.05, ***P < 0.001. **b** Heat map of dual cluster analysis: cytokine levels and mice. A horizontal label represents individual mice. *Cx43*^*fl/fl*^ mice are indicated in red and *Cx43* icKO mice are indicated in blue. The ranges for measured cytokine levels are shown in parentheses. *GM-CSF* granulocyte-macrophage colony-stimulating factor, *IFN* interferon, *IL* interleukin, *TNF* tumor necrosis factor

To elucidate the mechanism of remote suppressive effects by cortical astroglial *Cx43* ablation on spinal inflammation, cytokines/chemokines in the CSF from mice of both genotypes (*Cx43* icKO: *n* = 4, *Cx43*^*fl/fl*^: *n* = 4) were measured in the onset (dpi 10) phase of EAE by multiplexed fluorescence immunoassay. Two-way ANOVA revealed a significant difference in the expression levels of CSF cytokines according to genotype (*P* < 0.0001). Post-hoc analysis showed a significant suppression of IL-6, IFN-γ, and IL-10 in *Cx43* icKO mice compared with *Cx43*^*fl/fl*^ mice (*P* < 0.0001, = 0.0255, and < 0.0001, respectively) (Fig. 7 a). Dual cluster analysis of cytokines for each mouse demonstrated marked upregulation of almost all cytokines and chemokines examined in *Cx43*^*fl/fl*^ mice compared with *Cx43* icKO mice (Fig. 7 b). These findings indicate a remarkable suppression of pro- and anti-inflammatory cytokine secretion into the CSF in *Cx43* icKO mice, which may be partly responsible for the remote suppressive effects of anti-inflammatory A2 astroglia in the brain gray matter on spinal cord inflammation.

## Discussion

The novel findings of the present study are as follows. (1) *Cx43* ablation in brain gray matter astroglia attenuated acute and chronic EAE with reduced inflammatory demyelination in the spinal cord. (2) Brain gray matter astroglia-specific *Cx43* ablation enhanced A2 astroglial gene expression in the pre-immunized phase but attenuated the upregulation of proinflammatory A1 astroglial genes and markers at the onset and peak of EAE, respectively. (3) Brain gray matter astroglia-specific *Cx43* ablation promoted spinal cord microglia toward an anti-inflammatory phenotype at the peak of EAE. (4) CSF pro- and anti-inflammatory cytokine/chemokine levels were markedly decreased by brain gray matter astroglia-specific *Cx43* ablation at the onset of EAE. These findings collectively suggest a critical role of brain gray matter astroglia in regulating spinal cord inflammation via Cx43.

Two major subtypes of astroglia exist in the CNS: (i) GLAST^+^ astroglia residing in the gray matter, and (ii) GFAP^+^ astroglia residing in the white matter [27, 28]. During the maturation process, GLAST expression gradually diminishes in the spinal cord and its expression is confined to the brain gray matter in adults [29]. This is consistent with our observation in *Cx43* icKO mice, in which Cx43 expression was markedly diminished only in the brain gray matter, particularly the cerebellar and cerebral cortices, but not in the spinal cord.

In MOG-induced EAE in C57BL/6 mice, the spinal cord is most frequently and severely affected [30, 31]. This was also observed in *Cx43* icKO mice on a C57BL/6 background, which also showed inflammatory demyelination in the spinal cord but little pathology in the brain cortex. *Cx43* icKO mice had a markedly decreased infiltration of F4/80^+^ macrophages and Iba1^+^ activated microglia in spinal cord lesions, whereas CD3^+^ T cell infiltration was not changed compared with *Cx43*^*fl/fl*^ mice. This suggests that the attenuation of acute EAE in *Cx43* icKO mice is mainly attributable to the marked downregulation of macrophage/microglial inflammatory reactions in the CNS but not by the suppression of T cell invasion to the CNS. Indeed, MOG-specific T cell responses were unaltered in *Cx43* icKO mice and Cx43 expression patterns in the spleen and draining lymph nodes were similar between *Cx43* icKO and *Cx43*^*fl/fl*^ mice, although immunocytes such as T cells also express Cx43 [24, 25]. Thus, the ablation of *Cx43* in the brain gray matter astroglia mainly dampens macrophage/microglia responses in the spinal cord, which attenuates EAE pathology and manifestations.

Even in the pre-immunized phase, *Cx43* icKO spinal microglia showed the upregulation of anti-inflammatory genes and downregulation of proinflammatory genes including chemokine-related genes when compared with *Cx43*^*fl/fl*^ mice. Thus, brain gray matter astroglia-specific *Cx43* ablation renders spinal microglia anti-inflammatory in the pre-immunized state, which may dampen the proinflammatory activation of spinal microglia during EAE. Indeed, microglial circularity was decreased at the peak of EAE, together with an increased tendency of Arg-1 expression, which indicates a reduced activation status of *Cx43* icKO spinal microglia upon EAE insult. In particular, the suppression of *CCL2*, *5*, *7*, and *8* in the pre-immunized phase, and *CXCL9* in the peak phase, all of which are essential chemokines for macrophages and T cells [32], were responsible for the reduced infiltration of peripheral blood-borne macrophages and T cells in *Cx43* icKO mice compared with *Cx43*^*fl/fl*^ mice.

A key question is how *Cx43* deletion in brain gray matter astroglia distantly modulates spinal microglia in the pre-immunized state and during neuroinflammation. In *Cx43* icKO mice, the remarkable upregulation of A2-specific gene sets in brain astroglia was observed in the pre-immunized phase compared with *Cx43*^*fl/fl*^ mice, which was followed by attenuated A1-specific gene responses in the onset phase of EAE and a significant decrease in GFAP^+^ astroglia expressing a representative A1 marker, C3, in *Cx43* icKO mice compared with *Cx43*^*fl/fl*^ mice in the peak phase. These findings suggest that brain gray matter astroglia-specific *Cx43* ablation facilitates anti-inflammatory A2 astroglia differentiation in the pre-immunized state, which attenuates A1 astroglial activation in the onset and peak phases of EAE. We recently reported that oligodendroglia-specific *Cx47* ablation resulted in Cx43 hemi-channelization in astroglia, which showed remarkable A1 deviation [11]. These A1 astroglia promoted microglia toward a proinflammatory activation state, which aggravated EAE clinically and pathologically [11]. Our current *Cx43* ablation results appear to be the opposite of the *Cx47* ablation effects on neuroinflammation. This suggests that astroglial and oligodendroglial Cxs positively or negatively modulate CNS inflammation. In particular, brain gray matter astroglia might potentiate inflammation upon encephalitogenic challenge, which is reversed by the ablation of *Cx43*. Recently, the non-channel functions of Cx43 have attracted much attention. Cx43 molecules can bind to DNA and RNA and exert translational modulation on many genes [33, 34]. Thus, the ablation of *Cx43* may alter astroglial functional states via translational effects, which should be explored in future in vitro studies.

Regarding the mechanism of the remote effects of brain gray matter astroglia on spinal cord inflammation, cytokine/chemokine levels in CSF drawn in the onset phase of EAE were all markedly decreased in *Cx43* icKO mice compared with *Cx43*^*fl/fl*^ mice. Notably, the concentrations of IL-6 and IFN-γ were significantly suppressed by brain gray matter astroglia-specific *Cx43* ablation. Astroglia are an essential source of IL-6 in the CNS [35, 36] and they also produce IFN- γ upon activation [37]. IL-6 potentiates inflammation [35] by facilitating Th17 cell differentiation [38, 39] and augmenting the accumulation of pathogenic CD4^+^ T cells into inflammatory sites [31]. IFN-γ activates microglia toward a proinflammatory phenotype [40]. Therefore, these cytokines can exacerbate EAE. The downmodulation of these proinflammatory cytokines in *Cx43* icKO mice is likely the cause of the attenuation in EAE pathology. Conversely, another downregulated cytokine, IL-10, is a representative anti-inflammatory cytokine. However, IL-10 was also reported to be increased in the onset phase of EAE, as a host defense mechanism against neuroinflammation [41]. A decrease in IL-10 might reflect less severe neuroinflammation in *Cx43* icKO mice. These findings collectively suggest that the brain gray matter astroglia-specific ablation of Cx43 promotes astroglia toward an anti-inflammatory A2 phenotype, which inhibits proinflammatory A1 activation and results in reduced proinflammatory CSF cytokine/chemokine levels. This CSF cytokine/chemokine-mediated mechanism may be partly responsible for the remote anti-inflammatory effects on spinal cord inflammation. Thus, the activation of brain astroglia prior to the onset of EAE, as shown by in vivo imaging [18], is likely to promote the following overt inflammation in the spinal cord.

A major limitation of the present study was the lack of the above-mentioned in vitro studies of astroglia. Further in vitro functional assays of isolated astroglia and microglia will complement the present findings. In addition, a detailed in situ analysis of proinflammatory cytokines and chemokines in brain gray matter astroglia should be performed prior to the onset of EAE to further characterize the roles of these astroglia in neuroinflammation. Finally, there was no obvious inflammatory demyelination in the brain but there was a minimal increase of Iba1^+^ microglia in the hippocampus, which is consistent with previous reports [42, 43]. Also, no atypical EAE manifestations suggestive of cerebral or cerebellar involvement were found in *Cx43* icKO mice; however, detailed tests of cognitive function should be performed in the future.

## Conclusions

We conclude that brain gray matter astroglia exert proinflammatory effects distantly on spinal cord inflammation upon the induction of EAE. The ablation of Cx43 promotes brain gray matter astroglia toward an anti-inflammatory phenotype, even in the pre-immunized state, thereby suppressing the proinflammatory activation of spinal microglia partly through the reduction of CSF cytokine/chemokine levels. Our study suggests that brain gray matter astroglial Cx43 is a novel therapeutic target in MS.

## Supplementary Information

**Additional file 1: Supplementary Fig. 1.** Spinal cord and brain pathology in the pre-immunized phase and at the peak of acute EAE in *Cx43fl/fl* and *Cx43* icKO mice. **Supplementary Fig. 2.** Western blots for Cx43 in CNS tissues from *Cx43* icKO and *Cx43fl/fl* mice. **Supplementary Fig. 3.** Cx43 expression in peripheral immune cells and T cell responses to MOG in *Cx43* icKO and *Cx43fl/fl* mice. **Supplementary Fig. 4.** Real time RT-PCR for the up-regulated chemokine genes identified by GSEA analysis. **Supplementary Tab 1.** Antibodies used in this study.

## Data Availability

The gene array results were uploaded to the gene expression omnibus repository (accession number: GSE148932) hosted by the National Center for Biotechnology Information (https://www.ncbi.nlm.nih.gov/geo/query/acc.cgi?acc=GSE148932). The datasets used and/or analyzed during the current study are available from the corresponding author on reasonable request. All the antibodies used during this study are described in the main text and supplementary information files of the article.

## Abbreviations

ANOVA: Analysis of variance
APC: Allophycocyanin
ARRIVE: Animal research reporting of in vitro experiments
AUC: Area under the curve
BCA: Bicinchoninic acid
BrdU: 5-Bromo-2′-deoxyuridine
BSA: Bovine serum albumin
CCL: CC chemokine ligand
CD: Cluster of differentiation
CNS: Central nervous system
CSF: Cerebrospinal fluid
Cx: Connexin
CXCL: CXC chemokine ligand
DAB: Diaminobenzidine
DAPI: 4′,6-Diamidino-2-phenylindole
DEG: Differentially expressed gene
Dpi: Days post inoculation
EAE: Experimental autoimmune encephalomyelitis
ELISA: Enzyme-linked immunosorbent assay
ES: Enrichment score
FDR: False discovery rate
FITC: Fluorescein isothiocyanate
Fl: Flox
GFAP: Glial fibrillary acidic protein
GLAST: Glutamate aspartate transporter
GM-CSF: Granulocyte-macrophage colony-stimulating factor
GSEA: Gene-set enrichment analysis
HBSS: Hank’s balanced saline solution
icKO: Inducible conditional knockout
IFN: Interferon
IL: Interleukin
KB: Klüber-Barrera
LFB: Luxol fast blue
MBP: Myelin basic protein
MOG: Myelin-oligodendrocyte glycoprotein
MS: Multiple sclerosis
PBS: Phosphate-buffered saline
PCR: Polymerase chain reaction
PE: Phycoerythrin
PFA: Paraformaldehyde
ROI: Region of interest
Rpm: Revolutions per minute
RPMI: Roswell Park Memorial Institute
SEM: Standard error of the mean
TGF: Transforming growth factor
Th: Helper T
TNF: Tumor necrosis factor
Treg: Regulatory T
WT: Wild-type
Bps: Base pairs
X-gal: 5-Bromo-4-chloro-3-indolyl-β-d-galactoside
