# Supplementary material for: Brain gray matter astroglia-specific connexin 43 ablation attenuates spinal cord inflammatory demyelination
Source: J Neuroinflammation. 2021 Jun 5;18:126. doi: 10.1186/s12974-021-02176-1 (PMC8180177; doi:10.1186/s12974-021-02176-1)
Supplement: Supplementary file 1 — Additional file 1: Supplementary Fig. 1. Spinal cord and brain pathology in the pre-immunized phase and at the peak of acute EAE in Cx43fl/fl and Cx43 icKO mice. Supplementary Fig. 2. Western blots for Cx43 in CNS tissues from Cx43 icKO and Cx43fl/fl mice. Supplementary Fig. 3. Cx43 expression in peripheral immune cells and T cell responses to MOG in Cx43 icKO and Cx43fl/fl mice. Supplementary Fig. 4. Real time RT-PCR for the up-regulated chemokine genes identified by GSEA analysis. Supplementary Tab 1. Antibodies used in this study. [file 12974_2021_2176_MOESM1_ESM.pdf]

Supplementary Figure Legends

Supplementary Fig. 1 Spinal cord and brain pathology in the pre-immunized phase and at the peak of acute EAE in *Cx43$^{fl/fl}$* and *Cx43* icKO mice.

Paraffin sections of mouse spinal cord and brain samples from the pre-immunized phase and dpi 17 (the peak of acute EAE) are shown. For quantification, the regions of interest (ROI) were outlined by a yellow line for the spinal cord, and by a yellow square for the stratum radiatum of hippocampus, as indicated. (a) HE staining. Bars: 100 µm. (b) Klüver-Barrera (KB) myelin staining using Luxol fast blue (LFB). Bars: 100 µm. (c) Statistical analysis of LFB$^+$ area % in the ROI of each image. One-way ANOVA followed by Tukey's post-hoc analysis was performed. n = 4. **: $P < 0.01$, ***: $P < 0.0001$. (d) Anti-MBP immunostained images. Bars: 100 µm. (e) Statistical analysis of MBP$^+$ area % in the ROI of each image. One-way ANOVA followed by Tukey's post-hoc analysis was performed. n = 4. **: $P < 0.01$, ***: $P < 0.0001$. (f) Anti-Iba1 immunostained images. Bars: 100 µm. (g) Statistical analysis of Iba1$^+$ area % in the ROI of each image. One-way ANOVA followed by Tukey's post-hoc analysis was performed. n = 4. ***: $P < 0.0001$. (h) Anti-CD3 immunostained images. Bars: 100 µm.

Supplementary Fig. 2 Western blots for Cx43 in CNS tissues from *Cx43* icKO and *Cx43$^{fl/fl}$* mice.

(a) Western blots for Cx43 and β-actin in different CNS regions after tamoxifen treatment (n = 4). (b) Quantitative analysis of Cx43 protein amounts in each CNS region

by densitometry (n = 4). The graph shows the relative levels of Cx43 normalized against β-actin by densitometry. The statistical significance of differences between *Cx43*$^{fl/fl}$ and *Cx43* icKO mice was determined by unpaired *t*-tests. *$P < 0.05$.

Supplementary Fig. 3 Cx43 expression in peripheral immune cells and T cell responses to MOG in *Cx43* icKO and *Cx43*$^{fl/fl}$ mice.

(a) I Immunofluorescence for Cx43 in the spleen and inguinal lymph nodes. Scale bars: 100 µm. (b) Splenic T cell proliferation assayed by bromodeoxyuridine (BrdU) incorporation at different MOG_{35-55} concentrations (0, 2.5, 12.5, and 25 µg/ml) in *Cx43* icKO and *Cx43*$^{fl/fl}$ mice (n = 4 per group) at dpi 17. (c–f) Inflammatory cytokine levels in culture supernatants of splenocytes obtained at dpi 17, stimulated with different concentrations of MOG_{35-55} (0, 2.5, and 25 µg/ml), and measured using a multiplexed fluorescence immunoassay. All data are shown as the means ± SEM. Statistical analyses were performed by one-way ANOVA. No significant differences were found between *Cx43* icKO and *Cx43*$^{fl/fl}$ mice.

Supplementary Fig. 4 Real time RT-PCR for the up-regulated chemokine genes identified by GSEA analysis.

Real-time RT-PCR for *Ccl2*, *Ccl5*, *Ccl7*, and *Ccl8* using isolated microglia RNA from *Cx43* icKO and *Cx43*$^{fl/fl}$ mice in the preimmunized phase and at the peak of acute EAE (dpi 17). Microglia isolated from spinal cords of mice in the same experimental group

(n = 4) were pooled and subjected to RNA extraction. Genes of interest were compared with and are expressed as ratios relative to the reference gene (18S ribosomal RNA, *RN18s*). Reduced rates of expression are indicated by downward arrows on bars with the value of each reduction.

Suppl Fig. 1

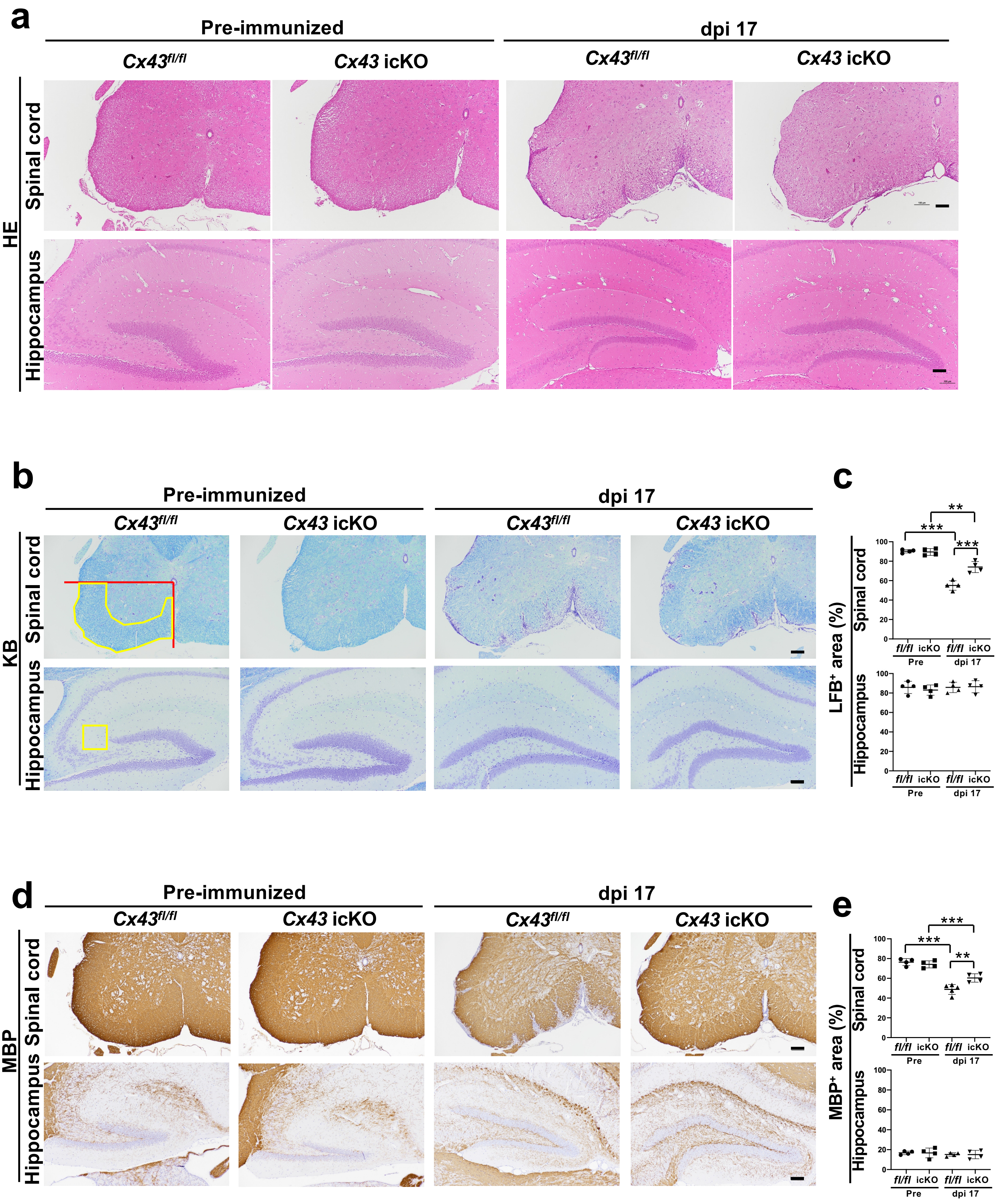
a
Pre-immunized
dpi 17
Cx43fl/fl
Cx43 icKO
HE
Spinal cord
Hippocampus
b
KB
c
LFB+ area (%)
fl/fl icKO fl/fl icKO
Pre dpi 17
**
***
d
MBP
e
MBP+ area (%)
0 20 40 60 80 100

Suppl Fig. 1 continued

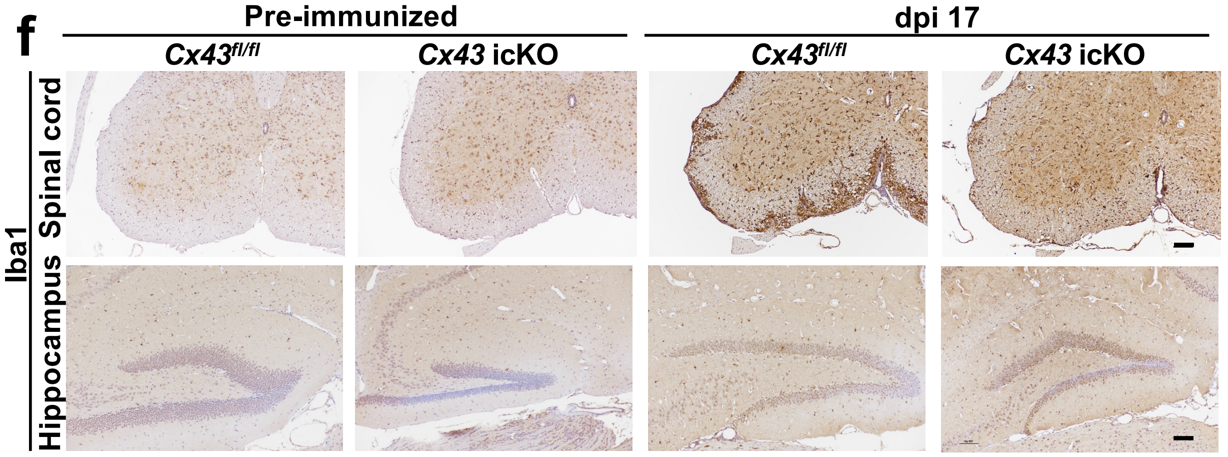

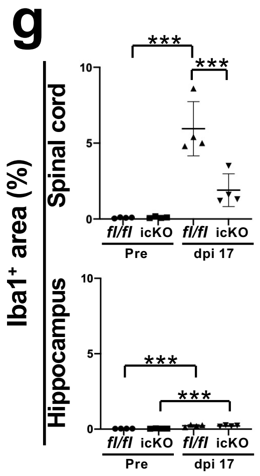

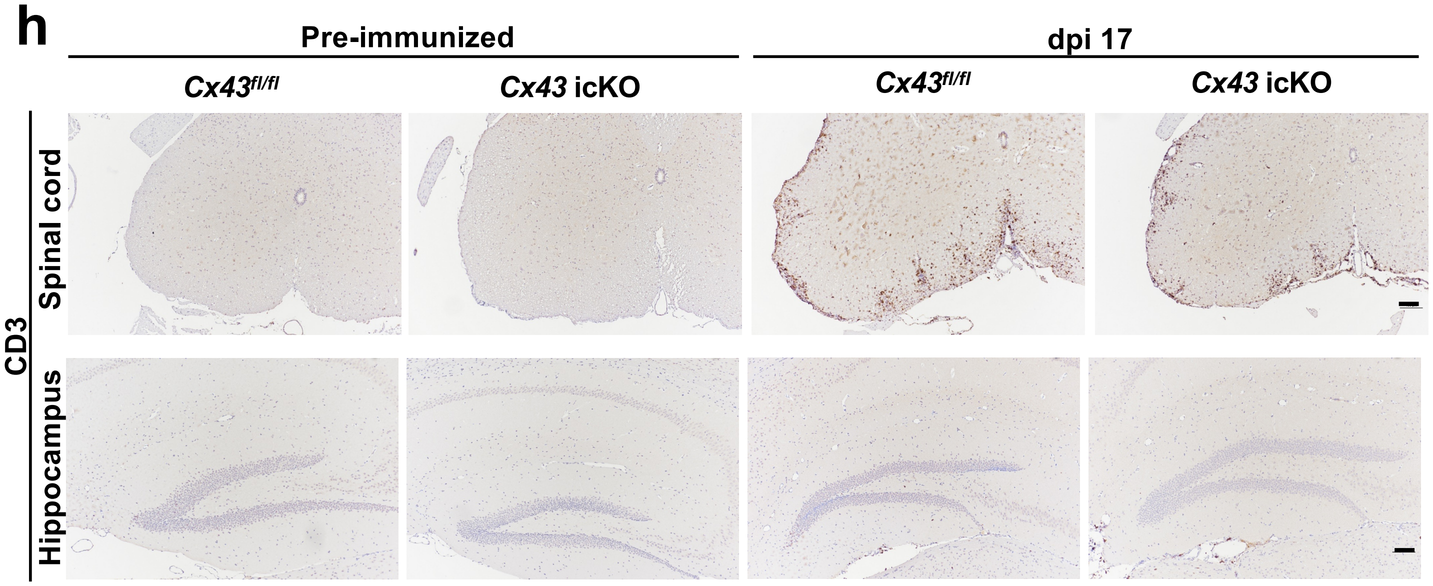

Suppl Fig. 2

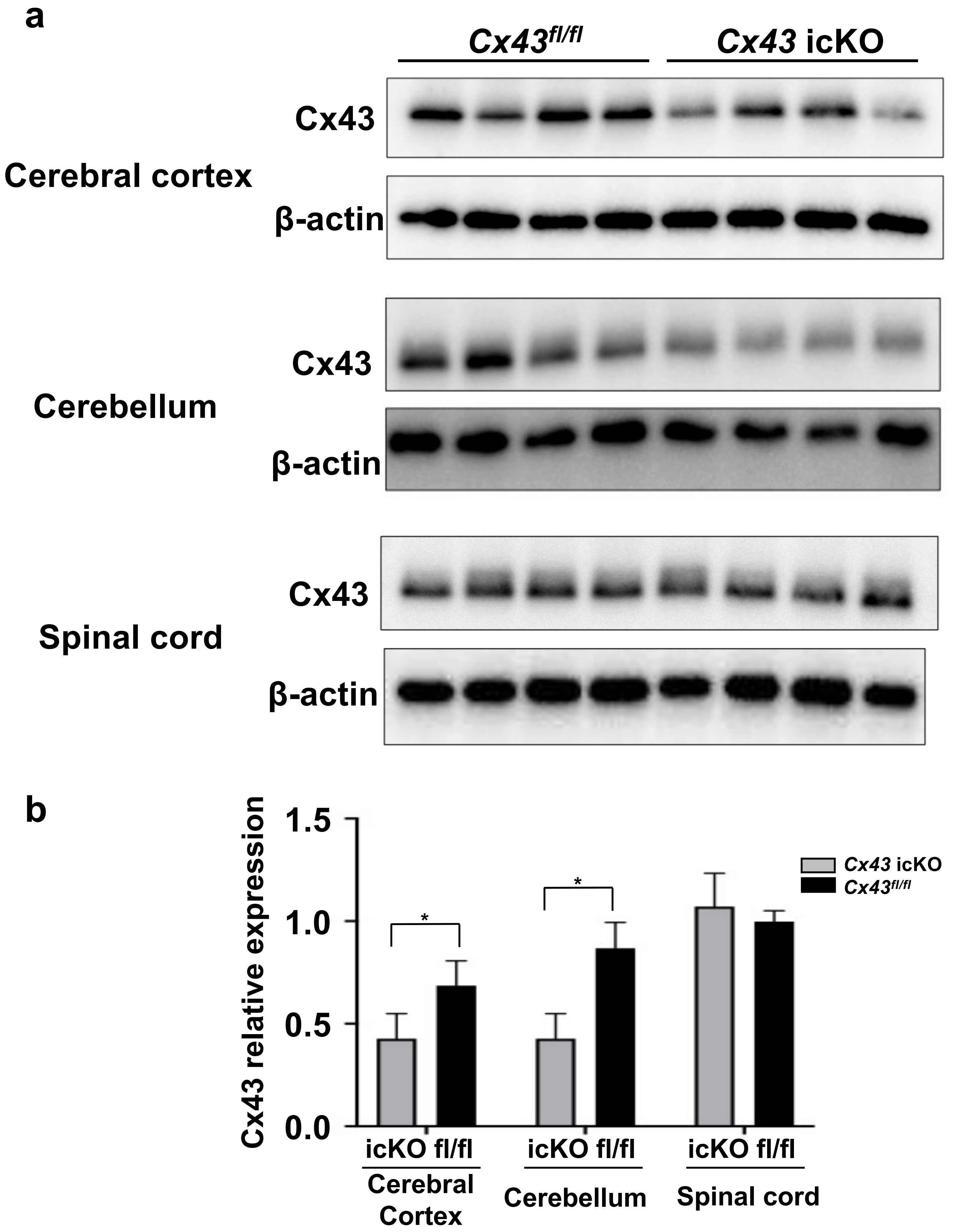
a
Cx43fl/fl
Cx43 icKO
Cx43
Cerebral cortex
β-actin
Cx43
Cerebellum
β-actin
Cx43
Spinal cord
β-actin
b
Cx43 relative expression
1.5
1.0
0.5
0.0
Cx43 icKO
Cx43fl/fl
*
*
icKO fl/fl
Cerebral Cortex
icKO fl/fl
Cerebellum
icKO fl/fl
Spinal cord

Suppl Fig. 3

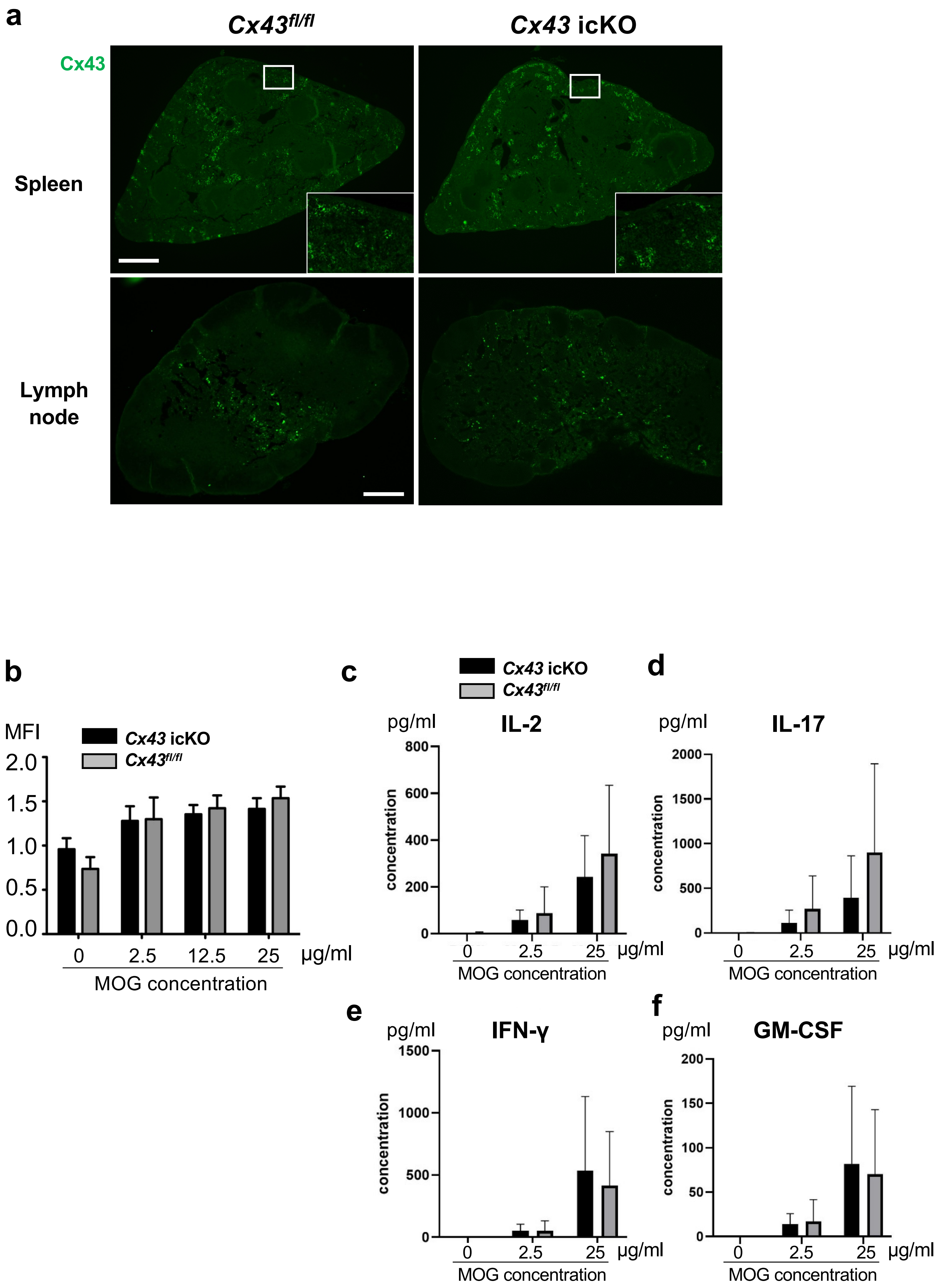

a
Cx43fl/fl
Cx43 icKO
Cx43
Spleen
Lymph node
b
MFI
Cx43 icKO
Cx43fl/fl
2.0
1.5
1.0
0.5
0.0
0
2.5
12.5
25
µg/ml
MOG concentration
c
Cx43 icKO
Cx43fl/fl
pg/ml
IL-2
concentration
800
600
400
200
0
0
2.5
25
µg/ml
MOG concentration
d
pg/ml
IL-17
concentration
2000
1500
1000
500
0
0
2.5
25
µg/ml
MOG concentration
e
pg/ml
IFN-γ
concentration
1500
1000
500
0
0
2.5
25
µg/ml
MOG concentration
f
pg/ml
GM-CSF
concentration
200
150
100
50
0
0
2.5
25
µg/ml
MOG concentration

Suppl Fig. 4

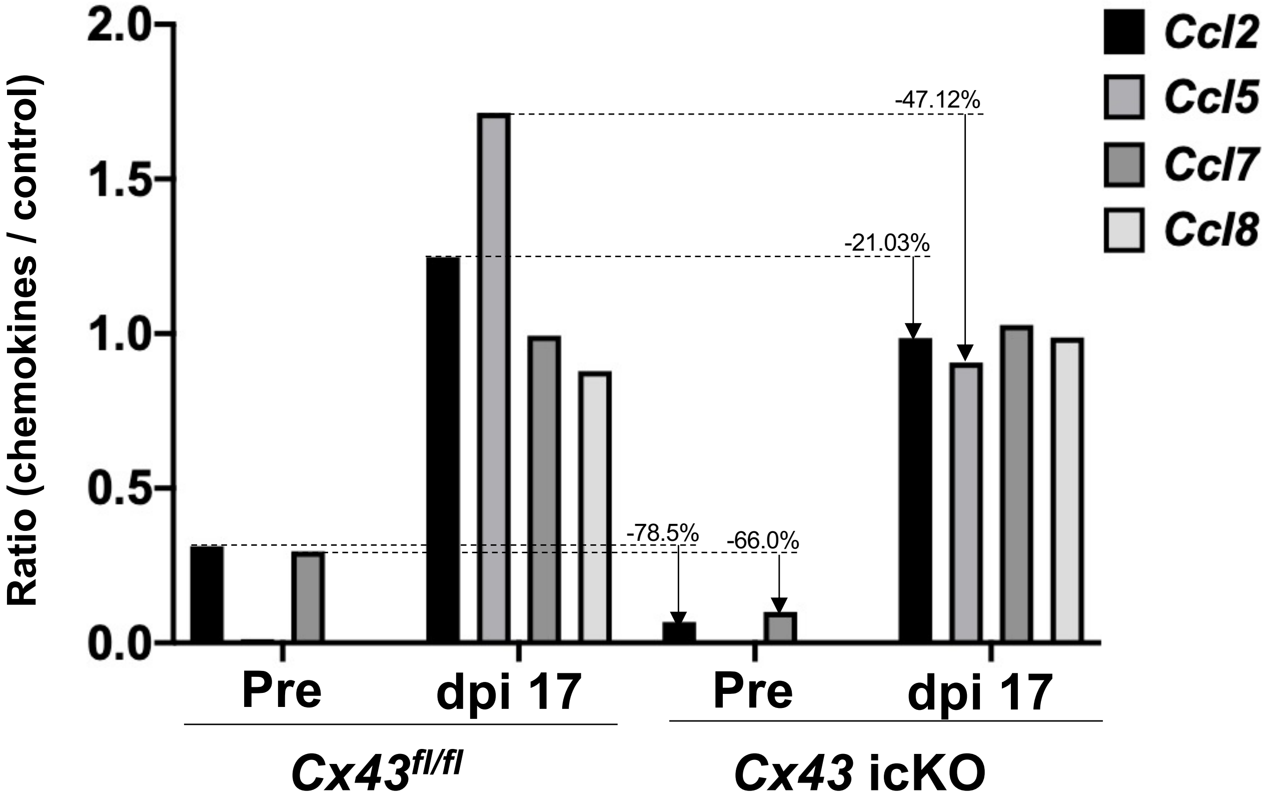

Ratio (chemokines / control)
2.0
1.5
1.0
0.5
0.0
Ccl2
Ccl5
Ccl7
Ccl8
-47.12%
-21.03%
-78.5%
-66.0%
Pre
dpi 17
Pre
dpi 17
Cx43fl/fl
Cx43 icKO

Supplementary Table 1. Antibodies used in this study.

Antigen	Clone	Type	Dilution	Incubation		Source
				Temp	Time	
**Connexin 43**	Polyclonal	Rabbit	1:1,000	4°C	Over night	Abcam
**Connexin 43 (immunoblot)**	Polyclonal	Rabbit	1:40,000	4°C	2 h	Abcam
**GFAP**	Polyclonal	Rabbit	Ready to use	4°C	Over night	DAKO
**Iba1**	Polyclonal	Rabbit	1:1,000	4°C	Over night	Wako
**Mouse CD3 molecular complex**	17A2	Rat IgG2b	1:200	4°C	Over night	BD Pharmin gen
**CD45**	IBL3/16	Rat IgG1	1:100	4°C	Over night	Bio-Rad AbD Serotec
**Myelin basic protein (MBP)**	1	Mouse IgG	1:1,000	4°C	Over night	DAKO

**S100a10**	Polyclonal	Goat	1:1,000	4°C	Over night	R&D Systems, Inc
**Complement component C3**	Monoclonal	Rat IgG2a	1:50	4°C	Over night	Hycult Biotech
**F4/80**	Cl:A3-1	Rat	1:100	4°C	Over night	Abcam
**Arginase 1 (Arg-1)**	Monoclonal	Mouse	1:100	4℃	Over Night	Santa Cruz
**β-actin**	AC-15	Mouse	1:10,000	4℃	1 h	Sigma

GFAP = glial fibrillary acidic protein.
